# Long-Term Activation upon Brief Exposure to Xanomleline Is Unique to M_1_ and M_4_ Subtypes of Muscarinic Acetylcholine Receptors

**DOI:** 10.1371/journal.pone.0088910

**Published:** 2014-02-18

**Authors:** Eva Šantrůčková, Vladimír Doležal, Esam E. El-Fakahany, Jan Jakubík

**Affiliations:** 1 Institute of Physiology Academy of Sciences of the Czech Republic, Prague, Czech Republic; 2 Department of Experimental and Clinical Pharmacology, University of Minnesota College of Pharmacy, Minneapolis, United States of America; Goethe University Frankfurt, Germany

## Abstract

Xanomeline is an agonist endowed with functional preference for M_1_/M_4_ muscarinic acetylcholine receptors. It also exhibits both reversible and wash-resistant binding to and activation of these receptors. So far the mechanisms of xanomeline selectivity remain unknown. To address this question we employed microfluorometric measurements of intracellular calcium levels and radioligand binding to investigate differences in the short- and long-term effects of xanomeline among muscarinic receptors expressed individually in Chinese hamster ovary cells. 1/One-min exposure of cells to xanomeline markedly increased intracellular calcium at hM_1_ and hM_4_, and to a lesser extent at hM_2_ and hM_3_ muscarinic receptors for more than 1 hour. 2/Unlike the classic agonists carbachol, oxotremorine, and pilocarpine 10-min exposure to xanomeline did not cause internalization of any receptor subtype. 3/Wash-resistant xanomeline selectively prevented further increase in intracellular calcium by carbachol at hM_1_ and hM_4_ receptors. 4/After transient activation xanomeline behaved as a long-term antagonist at hM_5_ receptors. 5/The antagonist N-methylscopolamine (NMS) reversibly blocked activation of hM_1_ through hM_4_ receptors by xanomeline. 6/NMS prevented formation of xanomeline wash-resistant binding and activation at hM_2_ and hM_4_ receptors and slowed them at hM_1_, hM_3_ and hM_5_ receptors. Our results show commonalities of xanomeline reversible and wash-resistant binding and short-time activation among the five muscarinic receptor subtypes. However long-term receptor activation takes place in full only at hM_1_ and hM_4_ receptors. Moreover xanomeline displays higher efficacy at hM_1_ and hM_4_ receptors in primary phasic intracellular calcium release. These findings suggest the existence of particular activation mechanisms specific to these two receptors.

## Introduction

Muscarinic receptors are members of the G protein coupled receptor (GPCR) family A. To date, five distinct subtypes of muscarinic acetylcholine receptors (M_1_–M_5_) have been cloned and sequenced [1]. Muscarinic acetylcholine receptors that are present both in the central and peripheral nervous systems are involved in numerous physiological and pathological processes and thus represent important pharmacological targets [2]. One of the most important roles of muscarinic receptor-mediated cholinergic neurotransmission in the CNS relates to cognitive functions, mainly through the activation of the M_1_ subtype of muscarinic receptors. Its disruption is connected with psychiatric and neurologic disorders including Alzheimer's disease (AD), Parkinson's disease, schizophrenia, epilepsy, sleep disorders, neuropathic pain, and others. Specifically, muscarinic agonists or inhibitors of acetylcholine esterase have been shown to reverse cognitive deficits associated with disrupted cholinergic neurotransmission in patients with a clinical diagnosis of Alzheimer presenile dementia [3] and a variety of other pathological states [4], [5].

However, subtype-selective muscarinic agonists are difficult to obtain due to high homology of the orthosteric agonist binding site among the five subtypes of muscarinic receptors. So far, one of the few known selective muscarinic agonists is xanomeline (3-hexoxy-4-(1-methyl-3,6-dihydro-2H-pyridin-5-yl)-1,2,5-thiadiazole) [6]. Xanomeline has been shown to stimulate phosphatidyl inositol hydrolysis in mice via M_1_ receptors [7]. In clinical studies xanomeline significantly improved cognition and ameliorated hallucinations and delusions in patients with Alzheimer's disease [8]. However, it was withdrawn from clinical trials due to unacceptable side effects including bradycardia, gastrointestinal distress, excessive salivation, and sweating [9]. Later on xanomeline proved to be also a potent agonist at M_4_ receptors [10], [11]. These findings have led to interest in xanomeline as a potential therapy for schizophrenia [12]–[15]. Besides its M_1_/M_4_ preference, xanomeline binds to all muscarinic receptor subtypes in a way that is resistant to intensive washing and causes persistent receptor activation or antagonism [16]–[22].

Functional subtype preference of xanomeline among muscarinic receptors is rather puzzling. Its reversible binding and receptor activation occur with the same affinity and potency at all subtypes of muscarinic receptors [20], [23], [24]. Also xanomeline wash-resistant binding occurs at all receptor subtypes with the same affinity [25]. So far, the only observed qualitative exception from uniform behavior of xanomeline at muscarinic receptors is functional antagonism by wash-resistant xanomeline at M_5_ receptors [22]. There are also differences in kinetics of xanomeline binding and activation between M_1_ and M_2_ receptors [20] and in long-term effects and receptor regulation between M_1_ and M_3_ receptors [24], [26].

In this study we investigated which property of xanomeline-receptor kinetics correlates with xanomeline functional preference for M_1_/M_4_ receptors observed *in vivo*. We focused on the differences among subtypes of muscarinic receptors in the formation of wash-resistant binding and persistent activation upon brief exposure to xanomeline followed by washing. To this end we employed radioligand binding and microfluorometric measurements of levels of intracellular calcium. Our results show commonalities of xanomeline reversible and wash-resistant binding and short-time activation but this commonality does not extend to long-term receptor activation. Wash-resistant xanomeline binding elicits full long-term receptor activation only at M_1_ and M_4_ receptors. Identification of this key difference is crucial for the design of future experiments aimed at unraveling the molecular mechanisms of xanomeline preference, with particular emphasis on identification of specific amino acid(s) or conformations associated with persistent activation by wash-resistant xanomeline unique to these two subtypes.

## Materials and Methods

### Cell culture

Chinese hamster ovary (CHO) cells stably expressing human variants of individual subtypes of muscarinic acetylcholine receptors were purchased from Missouri S&T cDNA Resource Center (Rolla, MO, USA). Cells were maintained in Dulbecco's modified Eagle medium enriched with 10% fetal bovine serum and 0,005% geneticin. For microfluorometry measurements about 250,000 cells were seeded on 24 mm diameter microscopic glasses (Karl Hecht KG, Sondheim, Germany) in 30 mm Petri dishes containing 3 ml DMEM and cultivated for 3 days. For binding experiments, 100,000 cells per well were seeded into 24-well plates in 2 ml of DMEM and grown for 4 days.

### Chemicals

Plasmid containing cDNA for human G protein G_16_ for transient transfection was from Invitrogen (Carlsbad, CA, USA). Other reagents for transient transfection – Lipofectamine and OptiMEM – were purchased from GibcoBRL (Gaithesburgh, MD, USA). Fura 2-AM for microfluorometry measurements was purchased from Molecular Probes – Invitrogen (Carlsbad, CA, USA). Fura 2-AM was dissolved in dimethylsulfoxide (Sigma, St. Louis, MO, USA) at 2 mM concentration and mixed 1∶1 with 20% pluronic P68 (Sigma). Krebs-HEPES buffer (KHB; final concentrations in mM: NaCl 138; KCl 4; CaCl_2_ 1.3; MgCl2 1; NaH_2_PO_4_ 1.2; Hepes 20; glucose 10; pH adjusted to 7.4) with or without probenecid (Serva Feinbiochemica, Heidelberg, Germany) was used for washing of cells. Forskolin and isomethylbutylxantine and muscarinic receptor ligands carbamoylcholine chloride (carbachol) and N-methylscopolamine bromide (NMS) were from Sigma (St. Louis, MO, USA) and xanomeline was from Eli Lilly & Company (Indianapolis, IN, USA). Radiolabeled muscarinic receptor antagonists methyl[^3^H]scopolamine ([^3^H]NMS) and quinuclidinylbenzilate ([^3^H]QNB) were from Amersham (Little Chalfont, Buckinghamshire, UK), radiolabeled adenine was from American Radiolabeled Chemicals (St. Louis, MO). Drugs were diluted directly in Krebs-HEPES buffer unless stated otherwise.

### Transient transfection

Using 6-well plates 5 µg of cDNA was diluted in 2.5 ml OptiMEM and 50 µl of Lipofectamine was diluted in 2.5 ml OptiMEM. After 5 mins of occasional stirring both solutions were combined (final concentration was 1 µg of cDNA and 10 µl of Lipofectamine per ml), stirred and then incubated 20 mins in room temperature and stirred occasionally. Meanwhile DMEM was removed from Petri dishes and cells were washed with 2 ml of sterile PBS. 0.8 ml of the mixture of cDNA-Lipofectamine was added to washed cells in each dish. After 6 hours incubation in 37°C 2 ml of warmed DMEM was added. After 48 hours cells were ready for the experiment.

### Fast microfluorometry

Microfluorometry experiments were carried out on the CHO cells stably expressing individual subtypes of muscarinic receptors on the third and fourth day after seeding. In order to facilitate measurements of calcium responses, cells stably expressing M_2_ and M_4_ receptors were one day after seeding transiently transfected with cDNA encoding human G protein G_16_ as described above. On the day of the measurement cells were twice washed with KHB then pre-labeled with 5 µM Fura 2-AM in KHB enriched with 1 mM pluronic for one hour at 37°C. After pre-labeling cells were washed twice with KHB, mounted to a superfusion chamber, placed on a stage of Olympus IX-90 inverted fluorescent microscope, application capillary was positioned at the edge of the view-field and suction capillary was positioned at the opposite edge of the view field less than 2 mm apart and continuously superfused at a flow rate 0.5 ml/min. The maximum possible volume of droplet between capillaries was 2 mm^3^. The measurements were conducted at room temperature air-conditioned to 27°C. The microscope was connected through a CCD camera to a computer equipped with Metafluor 2.0 software (Visitron Systems GmBH, Germany) for image acquisition and analysis. A cube with 330–385 nm excitation band pass and ≥420 nm emission wide band filter was used. Excitation wavelengths on Visitron monochromator were set to 340 nm and 380 nm. Acquisition time was 200 ms per image. Two acquisitions (pairs of images) were taken every second unless otherwise stated. During the measurements images of the whole visual field containing about 40 cells were saved and analyzed off-line after the measurements. Image darkest region devoid of cells was taken as the fluorescence background and was substracted from all values. Only cells responding to the first (control) carbachol stimulation were selected for further analysis. Eight to 12 cells with best response to first stimulation were selected (by exclusion of weakly and/or slow responding cells or cells with abnormal long-lasting response; the outliers in peak value, time to peak or fall time were identified by interquartile range (IQR) where data below Q1-1.5*IQR and above Q3+1.5*IQR were considered outliers) from every measurement and their calcium signals were averaged and normalized to basal calcium level. The average of initial 10-s period without agonist was taken as basal. Data were further analyzed by means of array oriented program Grace (plasma-gate.weizmann.ac.il/Grace/).

Four general schemes of calcium measurements were employed. In the first scheme differences among receptor subtypes in the long-term effects of brief exposure to xanomeline were tested. Initially, control stimulation with 300 nM carbachol lasting 5 s was performed. After 3 min of washing with KHB cells were stimulated with 10 µM xanomeline for 1, 3 or 10 min. Calcium levels in the absence of xanomeline were measured for the subsequent hour. At the end of measurement the second control stimulation with 300 nM carbachol for 5 s was carried out. Additional experiments with a slightly modified scheme were performed in order to evidence the differences between effects of wash-resistant xanomeline and the classical agonists carbachol, oxotremorine, and pilocarpine. In these experiments carbachol, oxotremorine, or pilocarpine were applied for one hour three minutes after an initial control 5-s stimulation with 0.3 µM carbachol and then washed in drug-free KHB for 30 min. At the end of measurement the second control stimulation with 0.3 µM carbachol for 10 s was carried out.

In the second scheme, effects of the antagonist NMS on delayed response to xanomeline were measured. After 5-s control stimulation with 300 nM carbachol cells were washed for 5 min with KHB and then stimulated with 10 µM xanomeline for 20 s. After 2-min of washing the cells were exposed for two min to 10 µM NMS and then they were washed again for another 4 min.

In the third scheme, effects of antagonist NMS on immediate response and formation of xanomeline wash-resistant receptor activation were probed. After initial 10-s control stimulation with 300 nM carbachol cells were washed for 5 min with KHB and then exposed for 3 min to 10 µM NMS. 10 µM xanomeline was applied for 1 min together with NMS during the second min of NMS treatment. Cells were finally washed for 3 min using drug-free KHB.

In the forth scheme, effects of extracellular calcium on xanomeline-induced oscillations of intracellular calcium were probed. After 5-s control stimulation with 300 nM carbachol cells were washed for 6 min with KHB Cells expressing M_1_ or M_4_ receptors were exposed for 3 min to 10 µM xanomeline and then washed with calcium-free KHB for additional 7 min.

### Binding experiments on membranes

For binding experiments 100,000 cells per well were seeded and grown in 3 ml of DMEM in 6-well plates. On day four after subculture cells stably expressing individual subtypes of muscarinic receptors from each well were detached by mild trypsinization, suspended in 1 ml of KHB, and then incubated at room temperature in KHB containing 10 µM xanomeline for 1, 3 or 10 min or in KHB containing 1 µM carbachol, 1 µM oxotremorine or 3 µM pilocarpine for 10 min. Control cells were sham treated with KHB. Subsequently, cells were spinned down and washed 3-times with 1 ml of ice cold KHB to remove free xanomeline and incubated in fresh KHB for another 10 min or one hour at room temperature. After incubation the cells were cooled on ice and membranes were prepared as follows. Treated cells were suspended in 1 ml of ice cold homogenization medium (100 mM NaCl, 10 mM MgCl_2_, 10 mM EDTA, 20 mM Na-HEPES pH = 7.4) and homogenized by two 30–second strokes at maximum speed and 30-second pause between strokes while cooled in ice by Ultra-Thurrax homogenizer. Homogenates were centrifuged at 1,000 g for 5 min and the resulting supernatant was centrifuged at 30,000 g for 30 min. Pellets were re-suspended in 1 ml of KHB and centrifugation was repeated. The membranes (50 µg of proteins per sample) were labeled with [^3^H]NMS in final concentration ranging from 60 pM to 4 nM at 30°C for 1 hour in 96-deep-well plates. Final incubation volume was 0.8 ml. Incubation was terminated by fast filtration through Whatman GF/C glass fiber filters on Brandel cell harvester. Non-specific binding was determined in the presence of 10 µM NMS. Filters were dried and then solid scintillator Meltilex A was applied using heating plate at 105°C for 75 s. After filters cooled radioactivity was measured in Microbeta scintillation counter (Wallac, Finland). Maximum binding capacity (B_MAX_) was corrected according to protein amount determined colorimetrically [27] on Wallac Victor 2 plate reader (Wallac, Finland).

### Assay of cyclic AMP formation

On day four after subculture cells stably expressing M_2_ or M_4_ subtypes of muscarinic receptors were suspended in KHB, preincubated for 1 h at 37°C with 0.25 µM [^3^H]adenine (10 µCi/ml). Xanomeline in a final concentration 10 µM was added to a portion of the cells for last 3 min of incubation. Cells were quickly washed three-times by centrifugation, resuspended in KHB and washed either for 10 min or 1 hour, centrifuged and washed twice by centrifugation and resuspended in KHB buffer containing 1 mM isobutylmethylxanthine and divided into individual incubation tubes. Forskolin was added to the cells at a final concentration of 5 µM or 20 µM. The incubation was in a volume of 0.8 ml per tube, with 300,000–400,000 cells per tube. Cells were incubated for 20 min at 37°C. Incubation was stopped by addition of 0.2 ml per tube of 2.5 M HCl and the extract was applied on a column filled with 1.5 g alumina. The column was washed with a portion of 2 ml of 100 mM ammonium acetate (pH 7.0) and the retained [^3^H]cAMP was eluted with the next portion of 4 ml of 100 mM ammonium acetate, collected in scintillation vials and quantified by liquid scintillation spectrometry. The synthesis of [^3^H]cAMP was measured as the difference between the content of [^3^H]cAMP in the samples at the end and in the beginning of the 20-min incubation period. Accumulation of [^3^H]cAMP in xanomeline-treated and sham-treated cells corrected for content of protein was compared.

### Data analysis

Data from binding experiments were pre-processed using Open Office (www.openoffice.org) and analyzed using Graph Pad Prism 5 (GraphPad Software Inc., La Jolla, CA, USA). Data from microfluorometry experiments were analyzed using Grace (Weizmann Institute of Science, Rehovot, Israel; http://plasma-gate.weizmann.ac.il/Grace/). Statistical analysis was done with statistical package R (www.r-project.org).

### Concentration response



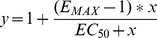
(Eq.1)where *y* is maximum stimulation by agonist at concentration *x,* E_MAX_ is maximal response and EC_50_ is half-efficient concentration.

### Saturation binding experiments



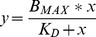
(Eq.2)where y is specific [^3^H]NMS binding at free concentration of [^3^H]NMS x, K_D_ is equilibrium dissociation constant and B_MAX_ is maximum binding capacity was fitted to the data from saturation binding experiments. Added radioligand was measured for each concentration by liquid scintillation and the initial concentration calculated based on specific radioactivity and final volume. Free radioligand concentration was calculated by subtraction of bound radioligand from initial radioligand concentration.

### Signaling efficacy

Apparent affinity constant K_G_ of the G protein for the agonist-receptor complex was calculated according Lu and Hulme [28] using the following equation:
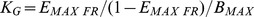
(Eq.3)where E_MAX FR_ is maximal response calculated according Eq. 1 and expressed as fraction of E_MAX_ of carbachol (E_MAX agonist_−1)/(E_MAX carbachol_−1) and B_MAX_ is maximum binding capacity calculated according Eq. 2 from binding data on cell membranes.

## Results

### Preliminary experiments

CHO cell lines expressed individual subtypes of muscarinic receptor in similar levels (Table S1 in File S1). In cells expressing hM_2_ or hM_4_ receptors and not transfected with G_16_ G protein the calcium response to 1 µM carbachol was weak (increase by 8 to 11% above basal level) and slow (time to reach maximum level was 50 to 80 s) (data not shown). Preliminary control experiments of the stability of intracellular calcium signal measured by the probe FURA-2 showed that the signal is stable (no photobleaching occurred) for more than 1 hour under experimental conditions (2 exposures for 200 ms every 20 s) and the response to carbachol was the same at 3 consequent stimulations with 3 min interval between stimulations as well as the stimulation after 1-hour superfusion (data not shown). Basal level signal was more than twice above the background level and peak signals (application of agonists) were about 20% of assay maximum (application of ionomycin). Intracellular calcium response to agonist carbachol and the partial agonists oxotremorine and pilocarpine was uniform among receptor subtypes (Fig. S1 and Table S3 in File S1).

### Potency and efficacy of brief exposure of cells to xanomeline on intracellular calcium level

**Figure 1:**
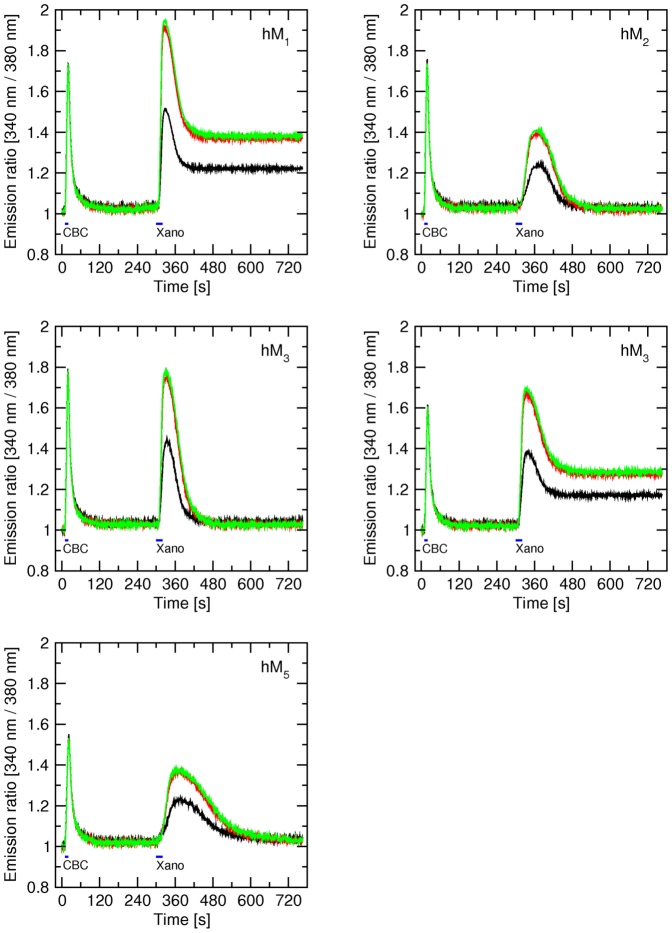
Concentration response to acute treatment with xanomeline. Cells were seeded, handled and loaded with Fura-2 as described in Methods. After an initial 10-s period cells were stimulated with 300 nM carbachol (CBC) for 5 s, washed with KHB for 5 min, then stimulated with 0.1 (black), 1 (red) or 10 µM (green) xanomeline (Xano) for 20 s and washed with KBH for 7 min. Traces are averages from 10 to 12 cells from representative experiment confirmed by 3 independent experiments. Signal variation (SD) among cells ranges from ±0.019 at the base line to ±0.035 at peaks. Parameters of calcium response are summarized in Table S2 in File S1. Calculated pEC_50_ and E_MAX_ of response to xanomeline are in Table 1.

Brief exposure (20 s) to xanomeline elicited a transient increase in intracellular calcium level (Fig. 1). At hM_2_, hM_3_ and hM_5_ receptors intracellular calcium level returned to basal but remained elevated at hM_1_ and hM_4_ receptors (Fig. 1). E_MAX_ effect elicited by 10 µM xanomeline was close to the maximal at all subtypes (Table S2 in File S1). Xanomeline had the same potency at all five receptor subtypes (Table 1). However, there was marked difference in xanomeline E_MAX_ among receptor subtypes. Calculated E_MAX_ is highest at hM_1_ and lowest at hM_5_ receptors (Table 1). Order of E_MAX_ values taken as per cent of full agonists carbachol E_MAX_ is M_1_>M_4_ = M_3_>M_5_>M_2_ and ranges from 90% to 44%. In control experiments (Fig. S1 in File S1) selectivity in efficacy of agonists oxotremorine and pilocarpine was much smaller and ranged from 56% at hM_2_ to 73% at hM_5_ to and from 52% at hM_2_ to 66% at hM_5_, respectively (Fig. S2 in File S1, Table 1). The order of apparent affinity constants of G-protein for agonist-receptor complex (K_G_) based on membrane expression level (Table 2) and calculated according Eq. 3 was M_1_>M_4_>M_3_>M_5_>M_3_ (Table 1).

**Table 1:** Potency and efficacy of agonists carbachol, oxotremorine, pilocarpine and xanomeline of intracellular calcium response.

	carbachol		oxotremorine		pilocarpine		xanomeline	
	pEC_50_	E_MAX_ [Emission ratio]	pEC_50_	E_MAX_ [Emission ratio]	pEC_50_	E_MAX_ [Emission ratio]	pEC_50_	E_MAX_ [Emission ratio]
**hM_1_**	6.92±0.07	2.04±0.08	7.33±0.06	1.65±0.05	7.02±0.05	1.65±0.05	7.1±0.1	1.94±0.08
**hM_2_**	6.71±0.05	2.26±0.09	7.19±0.05	1.71±0.06	6.95±0.07	1.66±0.05	7.1±0.1	1.42±0.04
**hM_3_**	6.80±0.06	2.20±0.09	7.33±0.05	1.68±0.06	6.97±0.05	1.68±0.06	7.1±0.1	1.78±0.06
**hM_4_**	6.65±0.07	2.06±0.08	7.17±0.06	1.64±0.05	7.15±0.07	1.60±0.05	7.1±0.1	1.70±0.06
**hM_5_**	6.81±0.05	1.85±0.07	7.29±0.05	1.62±0.05	7.00±0.05	1.56±0.05	7.1±0.1	1.37±0.04
			E_MAX_ [% of carbachol E_MAX_]	Efficacy [K_G_]	E_MAX_ [% of carbachol E_MAX_]	Efficacy [K_G_]	E_MAX_ [% of carbachol E_MAX_]	Efficacy [K_G_]
**hM_1_**			63	0.93	63	0.93	0.90	5.22
**hM_2_**			56	1.00	52	0.85	0.33	0.69
**hM_3_**			57	0.75	57	0.74	0.65	1.06
**hM_4_**			60	1.64	57	1.41	0.66	2.09
**hM_5_**			73	2.7	66	1.93	0.44	0.77

Potency is expressed as pEC_50_ (negative logarithm of half-efficient concentration), maximum increase E_MAX_ is expressed as ratio of emissions at 340 to 380 nm excitations and efficacy is expressed as per cent of carbachol E_MAX_ (maximum increase) and as G protein apparent affinity constant K_G_. Parameters were obtained by fitting equation Eq. 1 to the data from individual experiments in Fig. 1 (xanomeline) and the Fig. S1 in File S1 (carbachol, pilocarpine and oxotremorine) and subsequently by Eq. 3 using expression data from Table 2. Including Hill coefficient (slope factor) in the Eq. 1 does not improve the fit. Data are means±S.E.M. from 3 independent experiments.

**Table 2:** Maximum binding capacities (B_MAX_) of [^3^H]NMS binding to the membranes of the cells treated with xanomeline, carbachol, oxotremorine or pilocarpine are expressed as pmol of binding sites per mg of membrane protein.

	hM_1_	hM_2_	hM_3_	hM_4_	hM_5_
10-min washing					
control	1.80±0.03	1.29±0.03	1.75±0.03	0.928±0.023	0.998±0.021
xano 1-min	1.73±0.12	1.29±0.03	1.78±0.05	0.890±0.023	0.969±0.005
xano 3-min	1.86±0.12	1.34±0.02	1.79±0.09	0.898±0.097	0.973±0.035
xano 10-min	1.76±0.09	1.29±0.03	1.69±0.05	0.979±0.055	0.966±0.047
carbachol	1.34±0.02^*^	0.746±0.036^*^	1.34±0.01^*^	0.561±0.016^*^	0.802±0.012^*^
oxotremorine	1.54±0.12^*^	0.931±0.029^*^	1.46±0.02^*^	0.672±0.038^*^	0.833±0.035^*^
pilocarpine	1.64±0.02^*^	1.11±0.03^*^	1.62±0.02^*^	0.815±0.035^*^	0.941±0.013^*^
1-hour washing					
control	1.58±0.03^b^	1.22±0.02^b^	1.55±0.03^b^	0.775±0.021^b^	0.857±0.012^b^
xano 1-min	1.58±0.07^b^	1.23±0.05^b^	1.60±0.03^b^	0.818±0.033	0.866±0.014^b^
xano 3-min	1.64±0.05^b^	1.22±0.05^b^	1.46±0.07^b^	0.760±0.057^b^	0.843±0.040^b^
xano 10-min	1.54±0.03^b^	1.20±0.02^b^	1.61±0.03^b^	0.766±0.009^b^	0.862±0.012^b^
carbachol	1.31±0.02^*^	0.696±0.009^*b^	1.21±0.02^*b^	0.460±0.007^*b^	0.661±0.007^*b^
oxotremorine	1.54±0.09	1.11±0.05^*b^	1.36±0.12^*^	0.722±0.039	0.827±0.021
pilocarpine	1.60±0.09	1.16±0.03^*b^	1.58±0.07	0.761±0.029	0.838±0.035

Intact cells were exposed to 10 µM xanomeline for 1, 3 or 10 min or for 10 min to 1 µM carbachol, 1 µM oxotremorine or 3 µM pilocarpine or sham-treated (control) and washed with KHB for 10 min or 1 hour and then membranes were prepared as described in Methods. ^*^, different from control, ^a^, different from shorter treatment with xanomeline, ^b^, different from 10-min washing, P<0.05 by ANOVA and Tukey-Kramer post-test. Data are average values ± S.E.M. from 3 independent measurements performed in triplicates. Binding curves are in Fig. S3 in File S1.

### Immediate and delayed effects of brief exposure to xanomeline on intracellular calcium levels

**Figure 2:**
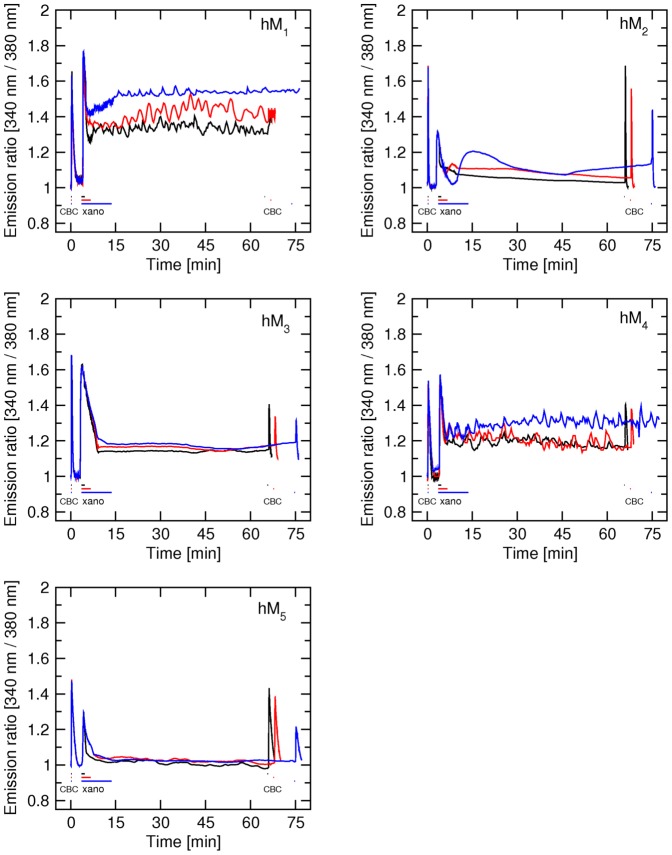
Effects of short-term application of xanomeline on the time-course of changes in intracellular calcium concentration in CHO cells expressing individual subtypes of muscarinic receptors. The time-course of intracellular calcium concentration (abscissa) after stimulation of hM_1_ to hM_5_ muscarinic receptor subtypes with the agonists carbachol (CBC) and xanomeline was measured as described in Methods. First stimulation: After 10 s of initial (resting) period 300 nM carbachol was applied for 10 s and then washed. Second stimulation: Three min after the first stimulation 10 µM xanomeline was applied for 1 min (black curve), 3 min (red curve) or 10 min (blue curve) followed by washing. Third stimulation: One hour after the second stimulation 300 nM carbachol was applied for 10 s followed by washing. Intracellular calcium concentration (ordinate) is expressed as fluorescence intensity (340 nm/380 nm) ratio normalized to basal calcium level. Representative traces are averages of 8 to 12 best responding cells from one experiment. Signal variation (SD) among cells ranges from ±0.017 at the base line to ±0.063 at peaks. Results were confirmed in 5 additional independent experiments. Parameters of xanomeline effects are summarized in Table S4 in File S1.

In microflourometric experiments of estimating the long-term effects of brief exposure to xanomeline on the level of intracellular calcium (Fig. 2) CHO cells expressing individual subtypes of muscarinic receptors were exposed to 10 µM xanomeline for 1, 3, or 10 min and intracellular calcium levels were measured for 1 hour under continuous superfusion with KHB to remove free xanomeline. Control 10-s stimulation with 300 nM carbachol was done before xanomeline application and at the end of measurements.

First (control) stimulation with 300 nM carbachol caused immediate mobilization of intracellular calcium at all subtypes of muscarinic receptors including hM_2_ and hM_4_ receptors (that were coupled to calcium response via transfection with the promiscuous G_16_ G protein α-subunit). After 4 mins of washing calcium levels returned to their basal values. Time needed to reach maximal response ranged from 6.2±0.3 s in case of M_2_ receptors to 7.9±0.7 s at hM_5_ receptors (Table S4 in File S1). The speed of calcium mobilization did not vary markedly among subtypes, but was slightly faster at hM_2_ than hM_5_ receptors. Thus, maximal calcium level elevation ranged from 1.47±0.04 to 1.68±0.09 fold of basal level at hM_5_ and hM_3_ receptors, respectively. It was the same at hM_1_, hM_2_ and hM_3_ and was higher at these subtypes than at hM_4_ and hM_5_ subtypes.

Stimulation with 10 µM xanomeline (lasting 1, 3 or 10 min) led to a fast increase in intracellular calcium at all muscarinic receptor subtypes. Unlike carbachol (control) stimulation, the speed of calcium mobilization and maximum calcium level elevation varied among subtypes. The response was fastest at hM_1_ receptors (time to reach maximum 9.6±1.7 s) and slowest at hM_5_ receptors (time to reach maximum 39±6 s). Xanomeline caused the strongest response at the hM_1_ receptor, increasing the calcium level to 118±3% of preceding control stimulation by carbachol. At hM_3_ and hM_4_ receptors the magnitude of response was the same as the response to carbachol (103±5 and 92±6% of response to carbachol, respectively). At hM_2_ and hM_5_ receptors the magnitude of xanomeline-induced calcium mobilization was about half of that induced by carbachol. After quickly reaching peak value intracellular calcium levels declined immediately despite ongoing xanomeline perfusion at all receptor subtypes. Cells expressing hM_1_, hM_3_ and hM_4_ receptors treated with xanomeline for 1, 3 or 10 min followed by washing showed increased calcium level after 60 min washing with KHB. At hM_2_ receptors, only 10-min xanomeline treatment increased calcium level after 60 min washing and at hM_5_ receptors calcium level returned to its original values even after 10 min xanomeline treatment. Elevated calcium levels at hM_1_ and hM_4_ receptors showed oscillations that did not appear at hM_2_ and hM_3_ receptors (Fig. 2).

Application of 300 nM carbachol for 5 s after exposure to xanomeline and washing still caused fast mobilization of intracellular calcium at all subtypes except for M_1_ (all treatments with xanomeline) and hM_4_ (10-min treatment with xanomeline) where calcium levels remained markedly increased after xanomeline stimulation. Xanomeline pretreatment followed by washing slowed down the speed of calcium mobilization and decreased the magnitude of the calcium signal by carbachol (Fig. 2; parameters are summarized in Table S4 in File S1). These effects were most prominent at hM_3_ receptors where time to reach maximum level was more than doubled and the maximal responses were close to half of the first stimulation.

### Effects of 1-hour exposure to the agonists carbachol, oxotremorine and pilocarpine on intracellular calcium level

**Figure 3:**
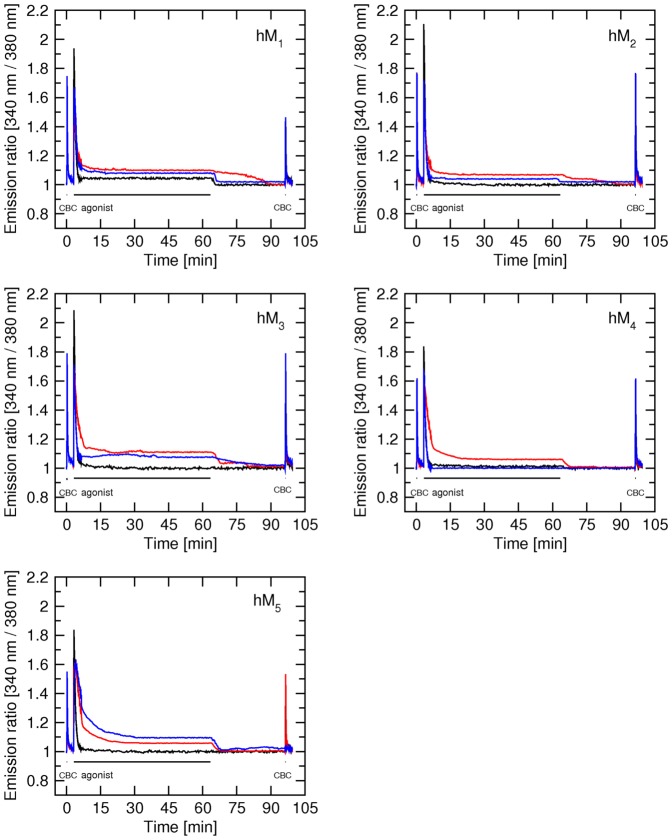
Effects of long-term application of classic agonists on the time-course of changes in intracellular calcium concentration in CHO cells expressing individual subtypes of muscarinic receptors. The time-course of changes in intracellular calcium concentration (abscissa) after stimulation of hM_1_ to hM_5_ muscarinic receptor subtypes with the agonists carbachol (CBC), oxotremorine and pilocarpine was measured as described in Methods. First stimulation: After 10 s of initial (resting) period 300 nM carbachol was applied for 10 s and then washed. Second stimulation: Three min after the first stimulation either 1 µM carbachol (black curve) or 1 µM oxotremorine (red curve) or 3 µM pilocarpine was applied for 1 hour followed by 30-min washing. Third stimulation: After washing following the second stimulation 300 nM carbachol was applied for 10 s followed by washing. Intracellular calcium concentration (ordinate) is expressed as fluorescence intensity (340 nm/380 nm) ratio normalized to basal calcium level. Representative traces are averages of 12 to 16 best responding cells from one experiment. Signal variation (SD) among cells ranges from ±0.018 at the base line to ±0.067 at peaks. Results were confirmed in 2 additional independent experiments. Parameters of agonist effects are summarized in Table S5 in File S1.

In microflourometric experiments measuring effects of long exposure to the agonists carbachol, oxotremorine and pilocarpine on the level of intracellular calcium (Fig. 3) CHO cells expressing individual subtypes of muscarinic receptors were exposed to 1 µM carbachol, 1 µM oxotremorine or 3 µM pilocarpine for 1 hour. Intracellular calcium levels were measured during agonist exposure and following 30-min of continuous superfusion with KHB. Control 5-s stimulation with 300 nM carbachol was done before agonist application and at the end of measurements.

First (control) stimulation with 300 nM carbachol caused immediate mobilization of intracellular calcium, similar to the effects of xanomeline (Table S5 in File S1). One-hour stimulation with 1 µM carbachol, 1 µM oxotremorine or 3 µM pilocarpine caused transient increase in intracellular calcium level. During 1-hour carbachol stimulation (Fig. 3, black traces) a transient increase in intracellular calcium level lasted about 3 min and returned to the basal level at all receptor subtypes except M_1_ where it remained slightly elevated (2.5% of peak value) until the end of carbachol stimulation. During oxotremorine stimulation (Fig. 2, red traces) intracellular calcium level transiently increased for about 4 min (hM_1_ and hM_2_), 5 min (hM_3_) or 15 min (hM_4_ and hM_5_ receptors). After this transient increase intracellular calcium level remained elevated until the end of stimulation. Steady increased levels of intracellular calcium ranged from 8% at M_5_ to 16% at M_1_ receptors. During pilocarpine stimulation (Fig. 3, blue traces) a transient increase in intracellular calcium was observed that in about 3 min returned to basal level (hM_2_ and hM_4_) or elevated level (hM_1_ and hM_3_). Elevated level at hM_1_ and hM_3_ receptors represented 10% and 14% of peak value of initial transient increase, respectively. In case of hM_5_ receptor the transient increase and return to the steady elevated level (16% of peak value) was slow and took about 30 min.

Immediately after 1-hour treatment with the agonists carbachol, oxotremorine and pilocarpine cells did not respond to 300 nM carbachol stimulation (data not shown). Response E_MAX_ to the third stimulation (300 nM carbachol) carried after 30-min washing with KHB (following 1-hour application of agonists) was diminished after carbachol treatment at all receptor subtypes (Fig. 3, Table S5 in File S1). Maximal response of the third stimulation was also diminished at hM_1_ receptors after oxotremorine and pilocarpine treatment. Response of hM_5_ receptors was completely abolished after pilocarpine treatment.

### Effects of xanomeline treatment on the number of membrane receptors

The number of membrane receptors was determined in [^3^H]NMS saturation binding of membranes prepared from cells treated with xanomeline for 1, 3 or 10 min (Fig. S3 A in File S1). To simulate conditions in microfluorometric experiments membranes were prepared 10 min or 1 hour after treatment of intact cells with xanomeline. Xanomeline treatment decreased the affinity of [^3^H]NMS to all receptor subtypes under every condition (Table 3) but did not change the number of membrane receptors at any receptor subtype under any condition (Table 2). Xanomeline-induced decrease in the affinity of [^3^H]NMS was largest at hM_4_ (25-fold decrease after 10-min treatment) and smallest at hM_2_ (2.5-fold decrease) receptors. In contrast, 10-min treatment of the cells with 1 µM carbachol, 1 µM oxotremorine or 3 µM pilocarpine (Fig. 3B) had no effect on [^3^H]NMS affinity at any receptor subtype (Table 3) but decreased the number of membrane receptors (Table 2). Carbachol decreased the number of membrane receptors by 20% at hM_5,_ about 25% at hM_1_ and hM_3_ and about 40% at hM_2_ and hM_4_ receptors. In general, oxotremorine and pilocarpine decreased the number of membrane receptors to a lesser extent. Extension of cell washing in KHB from 10 min to 1 hour led to a decrease in the number of membrane receptors even under control conditions (sham treatment without agonist). There was no change in the number of any of the receptor subtypes as a result of xanomeline treatment followed by washing for 1 hour. Treatment with carbachol reduced the number of membrane receptors by the same extent at all receptor subtypes except hM_1_ where 26% decrease in receptor number after 10-min washing fell to 17% after 1-hour washing. Similarly, the relative decrease in the number of membrane receptors (with respect to corresponding control) after oxotremorine treatment was smaller after 1-hour washing than after 10-min washing. There was no decrease in the number of membrane receptors after pilocarpine treatment followed by 1-hour washing. One-hour washing after treatment with carbachol, oxotremorine or pilocarpine had no effect on [^3^H]NMS affinity. Reduction in [^3^H]NMS affinity after 10-min treatment with xanomeline at hM_4_ receptors was the same after 10-min and 1-hour washing. Reduction in [^3^H]NMS affinity after 1-min and 3-min treatment with xanomeline at hM_4_ receptors became stronger during 1-hour washing. At hM_3_ receptors the reduction in [^3^H]NMS affinity became stronger during 1-hour washing. In contrast, the reduction in [^3^H]NMS affinity became weaker at the remaining receptor subtypes.

**Table 3:** Equilibrium dissociation constants (K_D_) of [^3^H]NMS binding to the membranes of the cells treated with xanomeline, carbachol, oxotremorine or pilocarpine is expressed in nM.

	hM_1_	hM_2_	hM_3_	hM_4_	hM_5_
**10-min washing**					
control	0.264±0.010	0.356±0.014	0.239±0.004	0.229±0.008	0.302±0.003
xano 1-min	2.11±0.10^*^	0.551±0.001^*^	0.717±0.021^*^	2.64±0.05^*^	1.44±0.02^*^
xano 3-min	2.44±0.18^*^	0.575±0.013^*^	0.863±0.037^*a^	3.67±0.36^*a^	1.79±0.04^*a^
xano 10-min	2.59±0.10^*^	0.894±0.030^*a^	0.899±0.017^*^	5.74±0.20^*a^	2.58±0.13^*a^
carbachol	0.255±0.006	0.348±0.015	0.226±0.003	0.235±0.004	0.294±0.008
oxotremorine	0.261±0.007	0.359±0.011	0.234±0.003	0.216±0.005	0.295±0.004
pilocarpine	0.248±0.006	0.384±0.014	0.236±0.004	0.217±0.004	0.288±0.011
**1-hour washing**					
control	0.248±0.006	0.367±0.009	0.232±0.003	0.220±0.011	0.312±0.006
xano 1-min	0.821±0.004^*b^	0.579±0.026^*^	1.34±0.01^*b^	3.45±0.15^*b^	1.08±0.01^*b^
xano 3-min	0.863±0.015^*ab^	0.589±0.019^*^	1.32±0.03^*b^	4.61±0.30^*ab^	1.45±0.02^*ab^
xano 10-min	0.915±0.014^*ab^	0.680±0.003^*ab^	1.67±0.09^*ab^	5.81±0.17^*a^	1.84±0.02^*ab^
carbachol	0.239±0.006	0.360±0.003	0.231±0.003	0.228±0.006	0.301±0.006
oxotremorine	0.241±0.004	0.365±0.007	0.226±0.009	0.227±0.005	0.297±0.009
pilocarpine	0.242±0.003	0.355±0.003	0.246±0.011	0.224±0.007	0.321±0.015

Intact cells were exposed to 10 µM xanomeline for 1, 3 or 10 min or for 10 min to 1 µM carbachol, 1 µM oxotremorine or 3 µM pilocarpine or sham-treated (control) and washed with KHB for 10 min or 1 hour and then membranes were prepared as described in Methods.^ *^, different from control, ^a^, different from shorter treatment with xanomeline, ^b^, different from 10-min washing, P<0.05 by ANOVA and Tukey-Kramer post-test. Data are average values ± S.E.M. from 3 independent measurements performed in triplicates. Binding curves are in Fig. S3 in File S1.

### Effects of blockade of the receptor orthosteric binding site on calcium level elevated by xanomeline

**Figure 4:**
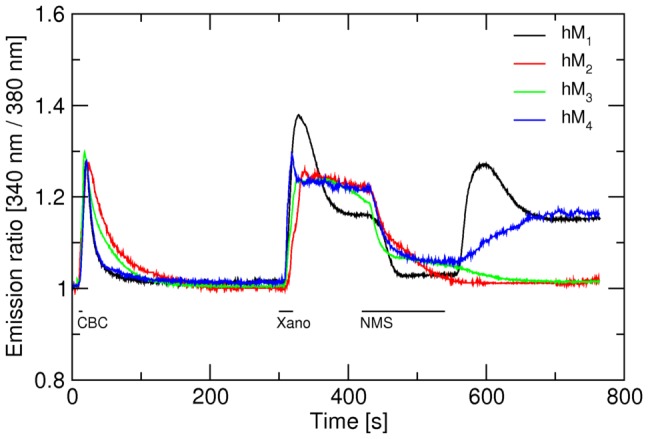
Effects of NMS on delayed elevation of intracellular calcium levels induced by short-term application of xanomeline at hM_1_ through hM_4_ receptors. Changes in the concentration of intracellular calcium (ordinate) are expressed as changes in fluorescence intensity (340 nm/380 nm) ratio normalized to basal calcium level. First (control) stimulation: 300 nM carbachol (CBC) for 5 s was applied. Second stimulation: At 300 s stimulation with 10 µM xanomeline (Xano) was applied for 20 s. After 2-min washing with KHB cells were superfused with 10 µM NMS for 2 min and then washed with KHB for additional 4 min. Traces are averages of 8 to 12 best responding cells from one experiment. Signal variation (SD) among cells ranges from ±0.015 at the base line to ±0.037 at peaks. Results were confirmed in 5 additional independent experiments. Parameters of xanomeline effects are summarized in Table S6 in File S1.

Prior to actual measurement of the effects of NMS on calcium levels elevated by xanomeline (Fig. 4) control stimulation by 300 nM carbachol for 5 s was done. After 5 min of washing with KHB, 20-s stimulation with 10 µM xanomeline was done. Cells were washed for two mins and then 10 µM NMS was applied for 2 min followed by washing in drug-free buffer to visualize the effects of xanomeline bound in a wash-resistant manner. Characteristics of immediate effects of carbachol and xanomeline on calcium responses (Table S6 in File S1) served as internal controls and were similar to those described above.

Application of 10 µM NMS brought increased calcium levels persisting after xanomeline exposure and washout to their basal levels at all subtypes. After switching back to perfusion with KHB calcium levels rose again at hM_1_ and hM_4_ but not at hM_2_ and hM_3_ receptors. In case of hM_1_ receptors an overshoot above steady state level appeared (Fig. 4, black trace, third peak). Time to reach maximum level after washing out NMS was several times shorter in case of the M_1_ receptor than in case of the hM_4_ receptor (Fig. 4, Table S6 in File S1). Increased steady state calcium levels after NMS withdrawal were similar at these two receptor subtypes and remained elevated during the following 1 hour of washing (not shown).

### Effects of NMS on formation of xanomeline wash-resistant activation

**Figure 5:**
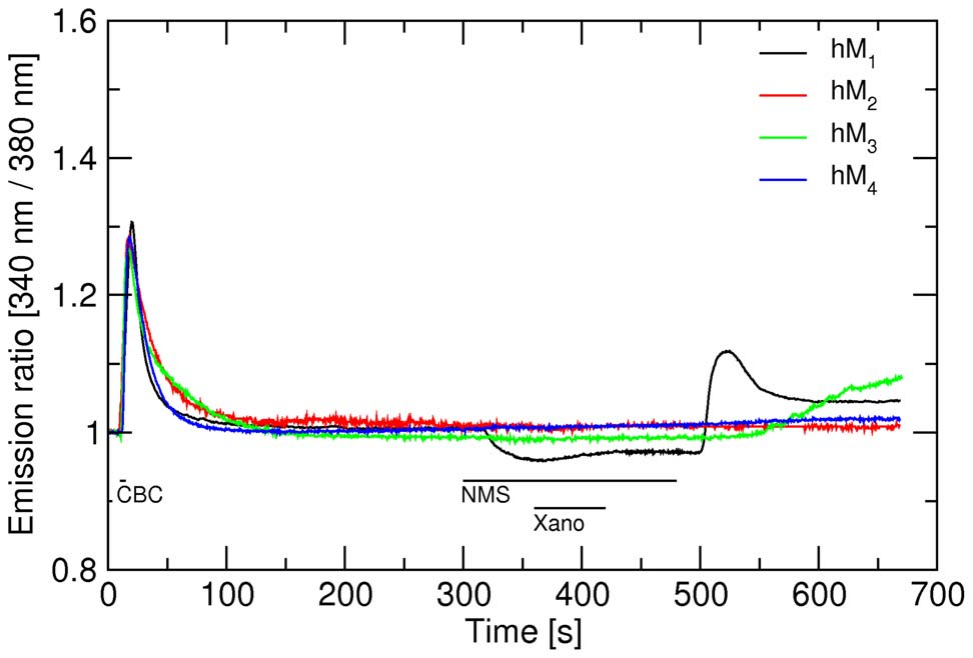
Effects of NMS on formation of xanomeline wash-resistant activation at hM_1_ through hM_4_ receptors. Changes in the concentration of intracellular calcium (ordinate) are expressed as changes in normalized fluorescence intensity (340 nm/380 nm) ratio normalized to basal calcium level. First (control) stimulation: Control 300 nM carbachol (CBC) was applied for 5 s. Second stimulation: At 5 min receptors were blocked by 10 µM of the antagonist NMS (1 min), then a mixture of 10 µM xanomeline (Xano) and 10 µM NMS was applied for 1 min and then 10 µM NMS was applied for an additional 1 min. Cells were then washed with KHB for additional 3 min. Representative traces are averages of 8 to 12 best responding cells from one experiment. Signal variation (SD) among cells ranges from ±0.015 at the base line to ±0.033 at peaks. Results were confirmed in 5 to 7 additional independent experiments. Parameters of xanomeline effects are summarized in Table S7 in File S1.

In another set of experiments the effects of the antagonist NMS on the formation of xanomeline wash-resistant receptor activation were investigated. Five mins after 5-s control stimulation with 300 nM carbachol, cells were superfused for 3 min with 10 µM NMS. Xanomeline was applied for 1 min at 10 µM (together with NMS) during the second min of NMS superfusion (Fig. 5, Table S7 in File S1).

NMS decreased basal level of calcium signal by 4.5% at hM_1_ receptors (Fig. 5, black trace) but did not cause any changes in intracellular calcium level at other receptor subtypes. Xanomeline applied concurrently with NMS had no immediate effect on calcium signal. However, removal of NMS during the final washing with fresh KHB (Fig. 5, from 480 s on) caused elevation of calcium level in cells expressing hM_1_ and hM_3_ receptors. Thus, NMS did not prevent formation of xanomeline wash-resistant binding at these subtypes and its removal unmasked activation by wash-resistant xanomeline. This unmasked activation persisted for the next 1 hour (not shown). A similar treatment protocol with xamomeline and NMS followed by washing did not restore activation of hM_2_ and hM_4_ receptors (Fig. 5, red and blue traces). Thus, NMS prevented the formation of xanomeline wash-resistant receptor activation at hM_2_ and hM_4_ receptor subtypes but not at hM_1_ and hM_3_ subtypes.

### Effects of NMS on formation of xanomeline wash-resistant action at hM_5_ receptors

**Figure 6:**
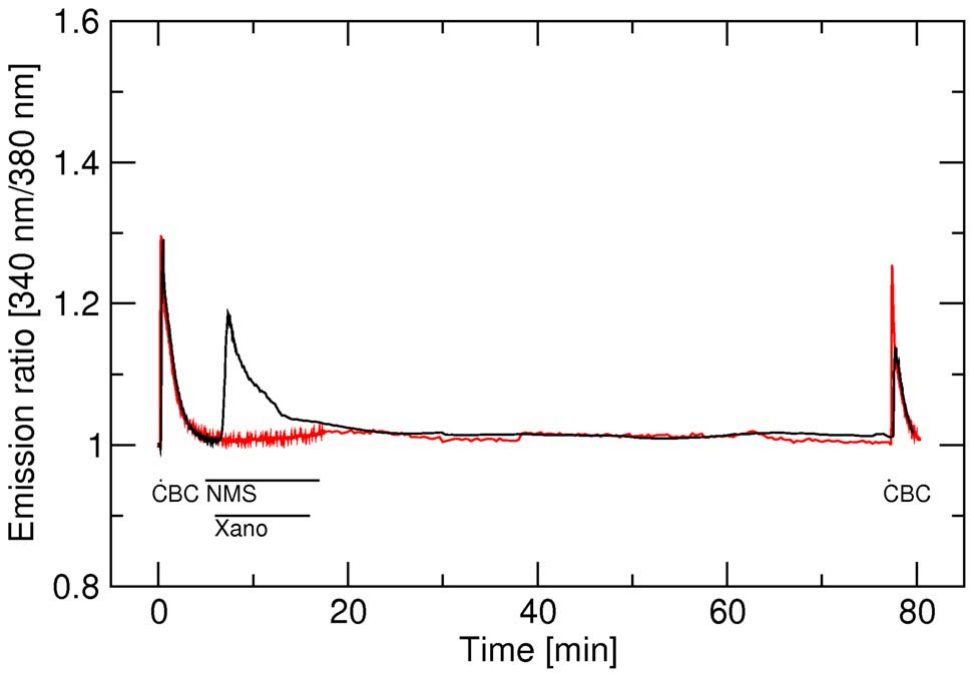
Effects of NMS on the formation of xanomeline wash-resistant action at hM_5_ receptors. Changes in the concentration of intracellular calcium (ordinate) are expressed as changes in normalized fluorescence intensity (340 nm/380 nm) ratio normalized to basal calcium level. Red trace: First stimulation: Control 5-s stimulation with 300 nM carbachol (CBC) was performed. Second stimulation: At 5 min receptors were blocked by 10 µM of the antagonist NMS (1 min), then a mixture of 10 µM xanomeline (Xano) and 10 µM NMS was applied for 10 min and finally 10 µM NMS wash- applied for an additional 1 min. Third stimulation: After washing of the cells with KHB for 60-min 300 nM carbachol was applied for 5 s. Black trace: Control curve, same as red one but NMS was not applied. Representative traces are averages of 8 to 12 best responding cells from one experiment. Signal variation (SD) among cells ranges from ±0.015 at the base line to ±0.032 at peaks. Results were confirmed in 5 additional independent experiments.

Effects of the antagonist NMS on the formation of xanomeline wash-resistant binding were tested in a separate set of experiments at M_5_ receptors since xanomeline did not produce long-term elevated calcium level at this receptor subtype under any experimental conditions. After control stimulation with 300 nM carbachol for 5 s and 5 min of washing with KHB cells expressing M_5_ receptors were treated with NMS and xanomeline in the same way as in the previous set of experiments, except that exposure to the mixture of xanomeline and NMS was extended to 10 min. Cells were then perfused with KHB for 1 hour and stimulated with 300 nM carbachol for 5 s (Fig. 6). The latter second stimulation led to slightly smaller and slower response compared to the control carbachol response (P<0.05 in paired t-test). This is in sharp contrast to the marked antagonism caused by wash-resistant xanomeline in the absence of NMS. These data indicate that NMS blocks the formation of xanomeline wash-resistant blockade of hM_5_ receptors.

### Lack of effects of changing extracellular calcium on calcium oscillations induced by xanomeline

**Figure 7:**
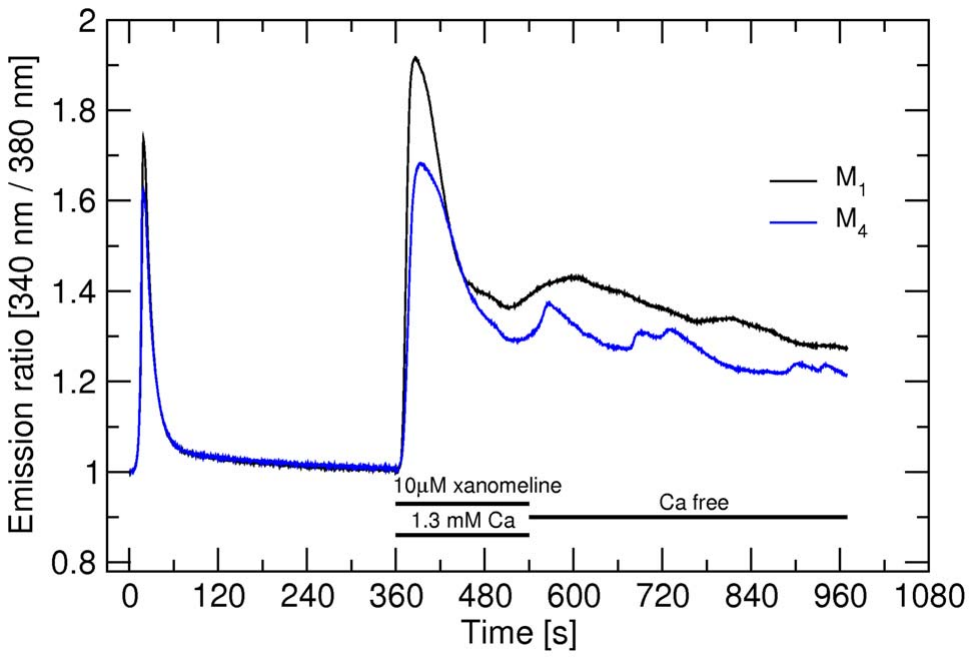
Lack of effects of changing extracellular calcium on calcium oscillations. Changes in the concentration of intracellular calcium (ordinate) are expressed as changes in normalized fluorescence intensity (340 nm/380 nm) ratio normalized to basal calcium level. Black trace: M_1_ receptors, blue trace: M_4_ receptors. First stimulation: Control 5-s stimulation with 300 nM carbachol (CBC) was performed. Second stimulation: After 6 min washing with KHB receptors were stimulated with 10 µM xanomeline for 3 min and then washed with calcium free KHB. Representative traces are averages of 12 best responding cells from one experiment. Signal variation (SD) among cells ranges from ±0.015 at the base line to ±0.063 at peaks. Results were confirmed in 2 additional independent experiments.

Regardless of the expressed subtype of muscarinic receptor CHO cells responded to 1 µM carbachol even in KHB where the concentration of calcium was lowered to 0.65 mM and even in calcium-free KHB (Fig. S4 in File S1). In reduced calcium KHB intacellular calcium peaks were lower than at normal calcium KHB and were even lower in calcium-free medium. Basal level of intracellular calcium was also reduced at the end of 12-min measurements. These data indicate that upon stimulation by carbachol calcium is released principally from intracellular stores and the decrease in peaks is likely due to depletion of intracellular stores. To test the possible role of extracellular calcium in xanomeline-induced oscillation in intracellular calcium at M_1_ and M_4_ receptors cells were stimulated for 3 min with 10 µM xanomeline and then washed with calcium-free KHB (Fig. 7). Washing cells with calcium-free KHB did not prevented oscillations in the intracellular calcium.

### Effects of xanomeline on accumulation of cAMP

**Figure 8:**
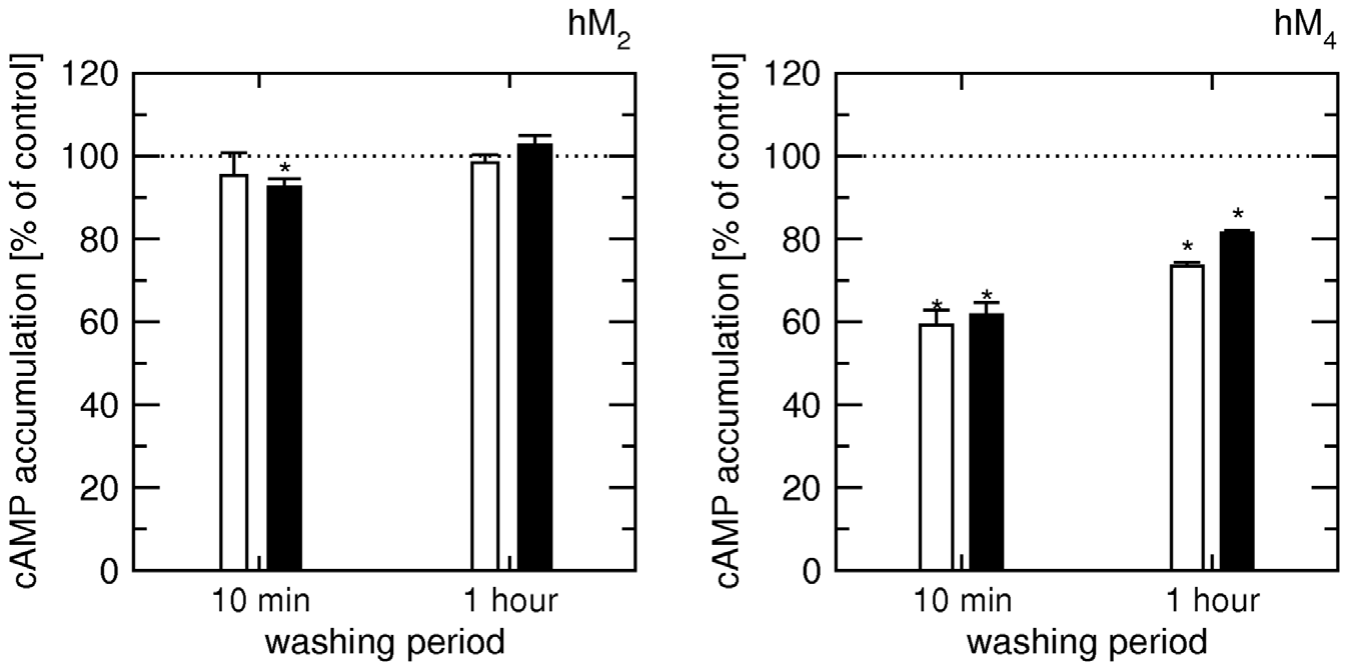
Effects of xanomeline on the accumulation of cyclic AMP. Cells expressing hM_2_ (left) or hM_4_ (right) receptors were treated with 10 µM xanomeline for 3 min followed by washing for 10 min or 1 hour (coordinate) prior to 20-min incubation with 5 µM (white bars) or 20 µM (black bars) forskolin. Accumulation of [^3^H]cAMP (ordinate) is expressed as per cent of control accumulation of [^3^H]cAMP in xanomeline sham treated cells (corrected for content of protein). Data are means ± S.E.M. from 3 experiments performed in triplicates. ^*^, different from control (sham treated) cells by t-test, P<0.05.

Accumulation of [^3^H]cAMP stimulated by 5 or 20 µM forskolin in cells expressing M_2_ or M_4_ receptors was measured after treatment of the cells with 10 µM xanomeline for 3 min followed by 10-min or 1-hour washing (Fig. 8). Xanomeline treatment had minimal effects on accumulation of [^3^H]cAMP in cells expressing M_2_ receptors under this experimental setup. After 10 min of washing xanomeline slightly (8%) inhibited [^3^H]cAMP accumulation (stimulated by 20 µM forskolin) but it had no effect on [^3^H]cAMP accumulation after 1-hour washing. In cells expressing M_4_ receptors xanomeline inhibited [^3^H]cAMP accumulation by almost 40% after 10-min washing and by more than 20% after 1-hour washing (Fig. 8).

## Discussion

The major finding of this study is that xanomeline functional preference for M_1_ and M_4_ muscarinic receptors originates at the receptor level. Xanomeline is one of few muscarinic agonits that is functionally preferring for M_1_ and M_4_ muscarinic acetylcholine receptors [7], [10]. Xanomeline exerts unusual pharmacological properties. Besides the reversible binding to and activation of muscarinic receptors it also binds to these receptors in a way that is resistant to intensive washing and is associated with persistent receptor activation [16]. Despite growing experimental data on the molecular mechanisms [19] and kinetics [20] of xanomeline binding and receptor activation, the basis of xanomeline functional preference remains enigmatic. Only indirect evidence from *in vivo* and behavioral experiments supports xanomeline selectivity [7], [10]. In contrast, xanomeline activates all subtypes of muscarinic receptors with the same potency [20], [23], [24] (Fig. 1 and Table 1), and the affinity of xanomeline reversible as well as wash-resistant binding is the same at all receptor subtypes [25]. So far, the only observed qualitative exception from uniform behavior of xanomeline at muscarinic receptors is its wash-resistant functional antagonism at M_5_ receptors [22]. The fundamental question where xanomeline selectivity *in vivo* comes from remains unanswered. Three possibilities may be considered, where xanomeline functional selectivity may be based on: a) pharmacodynamics (receptor level); b) differential receptor regulation (cell level) [24], [26]; c) pharmacokinetics (system level).

### Experimental setup

We employed fast microfluorimetric measurements of intracellular calcium levels that, unlike measurements of accumulation of second messengers (e.g. cyclic nucleotides or inositol phosphates), enabled us to observe potential fast short-term differences in the kinetics of receptor activation as well as long-term changes (both increase and decrease) in calcium signal reflecting potential differences in receptor activation and signal regulation. Only odd-numbered subtypes of muscarinic receptors directly elevate intracellular calcium levels via the G_q/11_ G proteins, phospholipase Cβ and 1,4,5-inositoltrisphosphate pathway. Even-numbered muscarinic receptors preferentially inhibit cAMP formation via G_i/o_ G proteins and changes induced in calcium level are slow and weak. To facilitate coupling of even-numbered receptors to the calcium-generating pathway we transiently transfected CHO cells with G_16_ G protein that links G protein coupled receptors to activation of phospholipase Cβ [29]. The coupling of hM_2_ and hM_4_ receptors was successful as evidenced by fast calcium response to carbachol that is similar to the response in odd-numbered subtypes (Fig. 1, Fig. S1, Table S3 in File S1). All five receptor systems responded to full non-selective agonist carbachol and partial agonists oxotremorine and pilocarpine in the same or very similar way proving the method to be applicable for detection of potential subtypes differences (Fig. S1, Table S3 in File S1). Moreover, xanomeline has the same affinity for all subtypes of muscarinic receptors and has similar potency at all these systems indicating the same coupling efficiency and no bias for xanomeline signal (Fig. 1, Table 1).

### Effects of acute exposure to xanomeline

Exposure to xanomeline for 20 s elicits a transient response in intracellular calcium (Fig. 1). The observed similar potency of xanomeline to release intracellular calcium at all receptor subtypes (Table 1) is in accordance with uniform xanomeline affinity for all receptor subtypes [25] and previous findings on functional responses to xanomeline [23]. However, xanomeline maximal response and coupling efficacy varied among subtypes. When maximal responses are expressed as per cents of the maximal response of the full agonist carbachol the rank order of maximal values follows putative xanomeline functional selectivity, being highest at hM_1_, intermediate at hM_3_ and hM_4_ and lowest at hM_5_ and hM_2_ receptors (Table 1). When receptor expression levels are taken into account and apparent affinity of G protein for agonist receptor complex K_G_ is calculated variations in xanomeline coupling efficacy become even more apparent (Table 1). In addition to higher maximal responses to xanomeline at hM_1_ and hM_4_ receptors, the calcium signal was longer lasting at these receptors compared to other subtypes (Fig. 1).Subtype differences in the coupling efficiency of xanomeline may thus be the basis of xanomeline functional selectivity. Coupling efficacy of oxotremorine and pilocarpine exhinits a different pattern from xanomeline and is highest at hM_5_ and lowest at hM_3_ receptors (Table 1). This excludes the possibility that coupling of hM_1_ and hM_4_ receptors to calcium signal is generally better in an agonist-independent manner.

### Sustained activation of M_1_ and M_4_ receptors

At the hM_1_, hM_3_, and hM_4_ subtypes, treatment with xanomeline as briefly as 1 min markedly elevated intracellular calcium, an effect that persisted for more than 1 hr after washing xanomeline (Fig. 2, black traces). In case of hM_1_ and hM_4_ receptors elevated calcium levels showed significant oscillation. Extended periods of calcium levels oscillating at levels higher than resting values indicate that these receptors are kept in an active conformation that overcome the efficiency of intracellular mechanisms responsible for sequestering free calcium. Lack of decrease in calcium level over extended period of time indicates that these receptors are not desensitized. Longer treatment with xanomeline was required to induce sustained elevated levels of intracellular calcium at hM_2_ receptors. At hM_5_ receptors xanomeline application induced only a transient increase in intracellular calcium concentration that depended on the length of treatment. The effects of the second application of carbachol were blocked by xanomeline treatment and washing at hM_1_, hM_4_ and hM_5_ receptors. While at M_1_ and hM_4_ receptors xanomeline behaved as a competitive agonist (no decrease in elevated calcium level) it behaved as competitive antagonist at hM_5_ receptors (no increase in calcium basal level). These data are in perfect fit with the observed functional preference of xanomeline for M_1_ and M_4_ receptors [7], [10], with delayed action of wash-resistant xanomeline at M_2_ receptors [20], [21] and functional antagonism by wash-resistant xanomeline at M_5_ receptors [22].

### Possible signal bias

Although bias of individual agonists towards different signaling pathway has been described at muscarinic receptors [30] it cannot be fully accountable for observed effects as M_1_ receptors couple to phospholipase Cβ via G_q/11_ G proteins while M_4_ receptors in our experiments couple via G_16_ G proteins. Importantly, intracellular calcium level during 1-hour treatment with carbachol is not substantially elevated at any receptor subtype but it is elevated during 1-hour treatment with the partial agonists oxotremorine and pilocarpine (Fig. 3). In contrast to the effects of xanomeline, the level of intracellular calcium upon treatment with these partial agonists was not significantly oscillating and was highest at M_5_ and M_3_ receptor. These observations rule out the possibility that high and oscillating levels of intracellular calcium after brief exposure to xanomeline is an artifact of M_1_ and M_4_ systems.

### Role of receptor regulation

Recent data suggest that xanomeline functional preference could be based on differential regulation of muscarinic receptor subtypes [24], [26]. It has been shown repeatedly that regulation of muscarinic receptors differs among receptor subtypes [31]–[33] and is agonist dependent [34]. Presumably, weaker and/or slower down-regulation of the signaling induced by xanomeline at one subtype could result in stronger signaling via this subtype over a prolonged period of time. Data in Tables 2 and 3 Fig. S3 in File S1, however, show that xanomeline (under our experimental conditions) forms wash-resistant binding and allosterically decreases affinity of NMS but does not cause internalization of any muscarinic receptors, unlike the full agonist carbachol and the partial agonists oxotremorine and pilocarpine (Tables 2 and 3 and Fig. S3 in File S1). Thus, sustained elevation of intracellular calcium level at only hM_1_ and hM_4_ receptors cannot be explained by different degrees of receptor internalization (to reduce xanomeline signal) and recycling (to gain responsiveness to carbachol). Sustained elevation of intracellular calcium level at only hM_1_ and hM_4_ receptors can neither be explained by higher degree of receptor desensitization at hM_2_ and hM_3_ as these receptors respond to agonist carbachol after activation by xanomeline better than hM_1_ and hM_4_ receptors.

### Role of kinetics

Our previous studies [20] showed that the kinetics of formation of xanomeline wash-resistant activation of hM_2_ receptors is much slower than that at hM_1_ receptors and suggested that differences in kinetics of wash-resistant binding and subsequent receptor activation may be involved in xanomeline functional preference. However, the kinetics of xanomeline wash-resistant binding does not correlate with the functional preference of xanomeline for M_1_ and M_4_ receptors. Although kinetics of wash-resistant binding is fastest at M_1_ receptors, it was equally fast at non-preferred M_5_ receptors and preferred M_4_ receptors (Table 2 and Fig. S3 A in File S1). Xanomeline wash-resistant binding further develops during 1-hour washing (Table 2 and Fig. S3 A (left vs. right) in File S1). Inhibition of NMS binding becomes weaker during 1-hour washing at preferred hM_1_ receptor and becomes stronger at non-preferred hM_3_ receptors (Table 3). Thus differential kinetics of xanomeline wash-resistant binding and activation cannot explain xanomeline preference for M_1_ and M_4_ activation.

### Agonist specific interactions

Other possible explanations of xanomeline functional preference include a differential mode of interaction with the receptor, interaction with different domains on the receptor or a different mode of receptor activation. For this purpose we tested whether xanomeline wash-resistant activation can be blocked by the orthosteric antagonist NMS (Fig. 4) and whether formation of xanomeline wash-resistant activation (Fig. 5) or wash-resistant functional antagonism (Fig. 6) can be blocked by NMS. As shown in Fig. 4, elevated calcium level in the continued presence of xanomeline was diminished by NMS at all subtypes (decrease at time 430 to 550 s). While intracellular calcium rises again after washing of NMS at hM_1_ and hM_4_ receptors it remains at basal level at hM_2_ and hM_3_ receptors (Fig. 4; Table S6 in File S1). Among these 4 receptor subtypes NMS has the slowest binding kinetics at hM_3_ receptors and the fastest at hM_2_ receptors [35]. Although slow binding kinetics of NMS at hM_3_ receptors can explain lack of increase in intracellular calcium after withdrawal of NMS at this receptor it contradicts with the fact that the decrease in calcium signal at this receptor after application of NMS is faster than at other subtypes, especially at hM_2_ where the kinetics of NMS is fastest. Lack of rise in intracellular calcium level after NMS withdrawal at hM_2_ receptors cannot be explained by binding kinetics of NMS (as NMS dissociation from hM_2_ is faster than from hM_1_ or hM_4_ receptors) and in agreement with Fig. 1 and Fig. 2 demonstrate that 20-s exposure of M_2_ receptors to 10 µM xanomeline is not sufficient for development of xanomeline wash-resistant activation.

When applied to receptors blocked by NMS xanomeline wash-resistant activation was reduced at hM_1_ and hM_3_ receptors (Fig. 5 black and green traces vs. Fig. 2 black traces; Table S7 vs. Table S4 in File S1) and completely blocked at hM_2_ and hM_4_ receptors (Fig. 5, red and blue traces). At hM_5_ receptor wash-resistant antagonism of xanomeline on activation by carbachol was diminished (Fig. 6). Thus, although to a different extent, NMS slows down the formation of xanomeline wash-resistant action at all receptors.

### The role of extracellular calcium

Absence of extracellular calcium does not affect muscarinic signaling indicating that persistent activation and oscillations observed at hM_1_ and hM_4_ receptors are not due to differential coupling to extracellular calcium influx at these subtypes. All cells responded well to carbachol even in calcium-free medium (Fig. S4 in File S1) demonstrating that the primary response to carbachol stimulation is independent from extracellular calcium. Similarly, washing the cells expressing hM_1_ or hM_4_ receptors with calcium-free KHB after xanomeline stimulation had no immediate effect on the prolonged increase in intracellular calcium and neither prevented calcium oscillations (Fig. 7). If this effect was due to extracellular (transmenbrane) calcium influx then removal of extracellular calcium would have immediate effects in reducing the calcium signal. Thus calcium oscillations observed only at hM_1_ and hM_4_ receptors are not due to coupling to extracellular calcium source. Taken together all five subtypes appear to couple to the same signaling pathway.

### Non-selective properties of xanomeline

In contrast with previous findings of uniform (non-selective) properties of xanomeline (i.e. the same affinity of both reversible and wash-resistant xanomeline binding at the various receptor subtypes [25] and potency of reversible xanomeline to activate all receptor subtypes (Fig. 1), numerous differences in xanomeline short and long-term effects on muscarinic receptors were found in the present study. They include differences in kinetics of xanomeline action, differences in NMS obliteration of xanomeline wash-resistant action and differences in interaction between xanomeline and NMS. However, none of these differences correlates with the observed functional preference of xanomeline for M_1_ and M_4_ receptors and thus cannot constitute the basis of xanomeline selectivity. The only principal difference among muscarinic receptor subtypes identified in this study that correlates with functional preference is variation in xanomeline efficacy at calcium signaling and the ability of wash-resistant xanomeline to keep M_1_ and M_4_ receptors in an active conformation over time. This is evidenced by persistent increase in intracellular calcium and, unlike at M_3_ receptors, inability of carbachol to induce further increase in calcium level. The physiological relevance of sustained hM_4_ receptor activation is supported by prolonged inhibition of accumulation of its natural second messenger cAMP that is absent at hM_2_ receptors (Fig. 8).

## Conclusions

Our results show uniform xanomeline potency in releasing intracellular calcium. In contrast, data demonstrate higher efficacy of xanomeline in calcium signaling and longer lasting responses at hM_1_ and hM_4_ receptors over the rest of the subtypes. Together, our data suggest the existence of a distinct activation mechanism at the hM_1_ and hM_4_ receptor subtypes.Taken together, the data presented herein answer the fundamental question of the origin of xanomeline selectivity observed *in vivo* and provide evidence that such preference is based on subtype differences in efficacy and long term activation and that is not due to differential receptor regulation at the cell level or in pharmacokinetic at a system level. However, further experiments are needed to delineate detailed molecular basis of xanomeline functional selectivity, most importantly the receptor domains involved.

## Supporting Information

File S1 **Portable document file containing results from control experiments and analytical data of** **Fig. 1** **through** **5** **of the main manuscript.**
(PDF)
