# Supplementary material for: Long-Term Activation upon Brief Exposure to Xanomleline Is Unique to M1 and M4 Subtypes of Muscarinic Acetylcholine Receptors
Source: PLoS One. 2014 Feb 18;9(2):e88910. doi: 10.1371/journal.pone.0088910 (PMC3928307; doi:10.1371/journal.pone.0088910)
Supplement: File S1 — Portable document file containing results from control experiments and analytical data of Fig. 1 through 5 of the main manuscript. (PDF) [file pone.0088910.s001.pdf]

Supporting information:

Long-term activation upon brief exposure to xanomleline is unique to M_1 and M_4 subtypes of muscarinic acetylcholine receptors

Eva Šantrůčková, Vladmír Doležal, Esam E. El-Fakahany, and Jan Jakubík

Summary

This supplementary information shows that all five cell lines used express muscarinic receptors at similar levels and that their response to the full agonist carbachol as well as the partial agonists oxotremorine and pilocarpine in elevation of intracellular calcium is uniform across receptor subtypes. Activation of all five subtypes leads to extracellular calcium independent release of calcium from intracellular stores. Thus the the calcium signal in this cellular model may serve for detection of potential subtype specific agonists. Xanomeline has the same binding affinity for all subtypes of muscarinic receptors and brief exposure to this drug has similar potency at all subtypes, indicating the same coupling efficiency and lack of system bias for the xanomeline signal. On the other hand different efficacies in elevation of intracellular calcium level at these cell lines indicates xanomeline functional selectivity among muscarinic receptor subtypes.

Methods

Methods were the same as in the main manuscript unless otherwise indicated.

Binding experiments on whole cells

Cells were seeded and grown in 24-well plates for each binding experiment independently. Subconfluent cells were washed twice with 1 ml of KHB and then incubated in KHB containing 10 µM xanomeline for 1, 3 or 10 min. Control cells were sham treated with KHB. Subsequently, cells were washed 3-times with 1 ml of KHB to remove free xanomeline and incubated in fresh KHB for another

10 min or one hour. After incubation the cells were cooled on ice and washed 3-times with 1 ml of KHB and then were labeled with 1 nM [^{3}H]NMS or [^{3}H]QNB. [^{3}H]NMS labeling lasted 20 min whereas labeling with [^{3}H]QNB was carried out overnight at 4°C. Non-specific binding was determined in the presence of 10 µM NMS or 10 µM QNB, respectively. After labeling cells were quickly washed 3-times with 1 ml of KHB and solubilized in 0.5 ml of 1M NaOH. Aliquots of 0.25 ml were mixed with 3 ml of Rotiszint liquid scintillator and radioactivity was measured in Microbeta scintillation counter (Wallac, Finland). Radioactivity was corrected according to protein amount determined colorimetrically on Wallac Victor 2 plate reader (Wallac, Finland).

Results

Receptor expression levels

Expression levels of muscarinic acetylcholine receptors were determined by binding of the radiolabeled antagonists [^{3}H]N-methylscopolamine ([^{3}H]NMS) and [^{3}H]-quinuclidinyl bezilate ([^{3}H]QNB). [^{3}H]NMS has a positive charge therefore does not penetrate membranes and in intact cells labels only receptors in the cell membrane. On the other hand, [^{3}H]QNB has no charge and is hydrophobic, therefore is able to penetrate membranes and labels extra- and intracellular receptors even in intact cells.

CHO cell lines used express muscarinic receptors at levels ranging from 29 to 111 fmol per mg of protein in membranes ([^{3}H]NMS binding) and from 24 to 184 fmol per mg of protein in total ([^{3}H]QNB binding (Table S1). One-hour washing with KHB (simulating conditions during microfluorometry) decreased the number of membrane as well as total receptors at all receptor subtypes, except for M_5 membrane receptors that remained unchanged.

Potency and efficacy of carbachol, oxotremorine and pilocarpine in changing intracellular calcium levels

CHO cells expressing individual subtypes of muscarinic receptors were stimulated with increasing concentrations of the full agonist carbachol or the partial agonists oxotremorine and pilocarpine and the level of intracellular calcium was measured by microflourometry. Potency (EC_{50}) and efficacy (E_{MAX}) were estimated by fitting Eq. 1 to the data from individual experiments. Numbers are means ± S.E.M. from 3 experiments.

Carbachol

The concentration of carbachol used was from 100 nM to 3 µM, stimulation lasted 5 s and cells were

washed for 6 min between stimulations (Fig. S1 A). The response to 3 µM carbachol was the same as to 1 µM carbachol indicating signal saturation (Table S3 A). Stimulation with 3 µM carbachol was repeated. The response to the second stimulation with 3 µM carbachol was smaller than the response to the first stimulation with the same concentration at all receptor subtypes indicating desensitization. To determine carbachol potency and efficacy the intracellular calcium response was measured for 3, 10 and 30 nM and 10 µM carbachol in similar experimental setup (Fig. S2 top). Carbachol potency was similar at all receptor subtypes (pEC_{50} from 6.92 ± 0.07 at M_1 receptors to 6.65 ± 0.07 at M_4 receptors) and efficacy (E_{MAX}) ranged from 2.26 ±0.09 at M_2 receptors to 1.85 ±0.07 at M_5 receptors (Table 1 in the main manusript).

Oxotremorine

The concentration of oxotremorine used was from 30 nM to 1 µM, stimulation lasted 10 s and cells were washed for 6 min between stimulations (Fig. S1 B). Prior to the first and after the last oxotremorine stimulation cells were stimulated with 300 nM carbachol for 5 s (non-desensitizing stimulation). The response to the last carbachol stimulation was the same as to the first indicating lack of desensitization to stimulation by oxotremorine (Table S3 B). The response to 300 nM and 1 µM oxotremorine was the same at M_1, M_3 and M_5 receptor subtypes indicating signal saturation at these subtypes. To determine oxotremorine potency and efficacy intracellular calcium response was measured for 1, 3 and 10 nM and 3 µM oxotremorine in similar experimental setup (Fig. S2 middle). Oxotremorine potency (pEC_{50}) ranged from 7.33 ± 0.06 at M_1 receptors to 7.17 ± 0.06 at M_4 receptors and efficacy was the same at all subtypes (E_{MAX} from 1.71 ±0.06 at M_2 receptors to 1.62 ±0.05 at M_5 receptors) (Table 1 in the main manuscript).

Pilocarpine

The concentration of pilocarpine used was from 100 nM to 3 µM, stimulation lasted 20 s and cells were washed for 8 min between stimulations (Fig. S1 C). Prior to the first and after the last pilocarpine stimulation cells were stimulated with 300 nM carbachol for 5 s (non-desensitizing stimulation). The response to the last carbachol stimulation was the same as to the first indicating lack of desensitization to stimulation by pilocarpine (Table S3 C). Response to 1 µM and 3 µM pilocarpine was the same at all receptor subtypes indicating signal saturation. To determine pilocarpine potency and efficacy intracellular calcium response was measured for 3, 10 and 30 nM and 10 µM pilocarpine in similar experimental setup (Fig. S2 bottom). Pilocarpine potency (pEC_{50} from 7.15 ± 0.07 at M_4 receptors to 6.95 ± 0.07 at M_2 receptors) and efficacy (E_{MAX} from 1.68 ±0.06 at M_3 receptors to 1.56 ±0.05 at M_5 receptors) were same at all subtypes.

Effect of agonists on [^{3}H]NMS binding to membranes

Exposure of the intact cells to 10 µM xanomeline for 1, 3 or 10 min does not cause receptor internalization as maximum binding capacity for [^{3}H]NMS remains unchanged (Fig. S3 A and Table 2 in the main manuscript). Only affinity of [^{3}H]NMS is decreased that may be explained by allosteric action of wash-resistant xanomeline on [^{3}H]NMS binding. Unlike xanomeline, 10 min exposure of the intact cells to 1 µM carbachol, 1 µM oxotremorine and 3 µM pilocarpine caused intermalization of all receptor subtypes as evidenced by decrease in maximum binding capacity for [^{3}H]NMS without change in affinity of [^{3}H]NMS (Fig. S3 A and Table 2 in the main manuscript).

Effect of changing the concentration of extracellular calcium

Cells were stimulated with 1 µM carbachol consecutively three-times for 5-s (Fig. S4). The first stimulation and subsequent washing were done under normal concentration of calcium (1.3 mM), the second one under reduced concentration of calcium (0.65 mM) and the third one in calcium-free medium. Cells responded in reduced calcium as well as calcium-free KHB at all receptor subtypes. This impliies that the source of receptor-induced elevation of cellular calcium is independent of extracellular sources, and is rather due to calcium release from the intracellular stores. Maxima of responses were slightly attenuated in reduced and calcium-free medium. This may be attributed to partial depletion of intracellular stores that cannot be replenished during and after stimulation at reduced calcium and calcium free conditions.

Tables

Table S1. Expression levels of individual subtypes of muscarinic receptors after 10-min and 1-hour incubation of the cells in KHB at room temperature.

	**10 min**		**1 hour**	
	^{3}H-NMS bindig [fmol / mg prot.]	^{3}H-QNB binding [fmol / mg prot.]	^{3}H-NMS binding [fmol / mg prot.]	^{3}H-QNB binding [fmol / mg prot.]
**hM_1**	37.0 ± 1.9	143 ± 7	28.8 ± 1.3*	107 ± 2*
**hM_2**	39.9 ± 4.8	48.1 ± 2.4	30.9 ± 0.9*	37.0 ± 3.3*
**hM_3**	111 ± 13	184 ± 7	89.9 ± 6.3*	162 ± 6*
**hM_4**	12.5 ± 0.6	23.9 ± 1.4	8.15 ± 1.20*	18.2 ± 0.7*
**hM_5**	29.5 ± 1.7	79.5 ± 4.8	19.6 ± 0.5	59.7 ± 1.8*

Expression level of muscarinic receptors is determined as ^{3}H-NMS (membrane receptors) and ^{3}H-QNB (all receptors) binding to intact cells and expressed in fmol of specifically bound radioligand per mg of protein. Data are averages ± S.E.M. from 4 to 8 independent experiments performed in triplicates. *, significantly different from 10-min treatment, $P<0.05$ by t-test, data from individual experiments paired.

Table S2. Parameters of calcium level changes upon acute exposure to xanomeline in Fig. 1 in the main manuscript.

	0.1 µM xanomeline		1 µM xanomeline		10 µM xanomeline	
	TTM [s]	E_{MAX} [Emission ratio]	TTM [s]	E_{MAX} [Emission ratio]	TTM [s]	E_{MAX} [Emission ratio]
hM_1	12 ± 1*	1.52 ± 0.04*	9.5 ± 0.8*	1.91 ± 0.06	9.1 ± 0.8	1.94 ± 0.07
hM_2	39 ± 8*	1.24 ± 0.03*	29 ± 7	1.39 ± 0.04	29 ± 6	1.41 ± 0.04
hM_3	15 ± 4*	1.44 ± 0.04*	11 ± 3	1.75 ± 0.06	11 ± 3	1.78 ± 0.06
hM_4	16 ± 3*	1.38 ± 0.03*	14 ± 2	1.67 ± 0.05	13 ± 2	1.70 ± 0.06
hM_5	47 ± 8*	1.23 ± 0.02*	39 ± 7	1.37 ± 0.03	35 ± 7	1.37 ± 0.03

Parameters are derived from experiments shown in Fig. 1 of the main manuscript. Data are means ± S.E.M. from 4 independent experiments; TTM, time to reach the maximum; *, different from 10 µM xanomeline by Dunnett's test, $P<0.05$.

Table S3. Parameters of calcium level changes upon exposure to agonists in Fig. S1.

Carbachol

	100 nM		300 nM		1 µM		3 µM (1st)		3 µM (2nd)	
	TTP [s]	E_{MAX}	TTP [s]	E_{MAX}	TTP [s]	E_{MAX}	TTP [s]	E_{MAX}	TTP [s]	E_{MAX}
M_1	5.9 ± 0.5	1.45 ± 0.03	6.0 ± 0.5	1.75 ± 0.03	6.2 ± 0.5	1.95 ± 0.04[a]	6.4 ± 0.5	2.02 ± 0.04	6.3 ± 0.6	1.92 ± 0.04*
M_2	4.0 ± 0.4	1.40 ± 0.03	4.1 ± 0.5	1.77 ± 0.03	4.2 ± 0.4	2.12 ± 0.04*	4.2 ± 0.5	2.20 ± 0.04	4.2 ± 0.4	2.06 ± 0.04*
M_3	4.4 ± 0.5	1.46 ± 0.03	4.5 ± 0.5	1.79 ± 0.04	4.6 ± 0.5	2.07 ± 0.04*	4.6 ± 0.5	2.14 ± 0.04	4.5 ± 0.4	2.07 ± 0.04*
M_4	4.5 ± 0.5	1.32 ± 0.03	4.5 ± 0.5	1.62 ± 0.03	4.7 ± 0.5	1.91 ± 0.04*	4.9 ± 0.5	1.97 ± 0.04	4.8 ± 0.5	1.82 ± 0.04*
M_5	4.4 ± 0.5	1.33 ± 0.03	4.5 ± 0.5	1.55 ± 0.03	4.6 ± 0.5	1.73 ± 0.03*	4.8 ± 0.5	1.82 ± 0.04	4.8 ± 0.5	1.73 ± 0.03*

Oxotremorine

	300 nM CBC (1st)		30 nM		100 nM		300 nM		1 µM		300 nM CBC (2nd)	
	TTP [s]	E_{MAX}	TTP [s]	E_{MAX}	TTP [s]	E_{MAX}	TTP [s]	E_{MAX}	TTP [s]	E_{MAX}	TTP [s]	E_{MAX}
M_1	6.2 ± 0.5	1.75 ± 0.03	10 ± 1	1.27 ± 0.03	9.9 ± 0.7	1.46 ± 0.03*	10 ± 1	1.56 ± 0.03*	10 ± 1	1.62 ± 0.03	6.2 ± 0.5	1.74 ± 0.04
M_2	4.2 ± 0.4	1.76 ± 0.03	7.8 ± 0.6	1.23 ± 0.03	8.1 ± 0.6	1.42 ± 0.03*	8.2 ± 0.7	1.58 ± 0.03*	8.3 ± 0.7	1.66 ± 0.03*	4.1 ± 0.4	1.76 ± 0.03
M_3	4.6 ± 0.5	1.79 ± 0.03	9.1 ± 0.7	1.27 ± 0.03	9.3 ± 0.7	1.46 ± 0.03*	9.3 ± 0.7	1.57 ± 0.03*	9.4 ± 0.7	1.65 ± 0.03	4.6 ± 0.5	1.80 ± 0.04
M_4	4.4 ± 0.5	1.63 ± 0.03	8.8 ± 0.6	1.21 ± 0.03	8.8 ± 0.7	1.37 ± 0.03*	9.0 ± 0.7	1.50 ± 0.03*	9.0 ± 0.7	1.59 ± 0.03*	4.3 ± 0.5	1.63 ± 0.03
M_5	4.6 ± 0.5	1.55 ± 0.03	9.2 ± 0.7	1.22 ± 0.03	9.3 ± 0.7	1.37 ± 0.03*	9.3 ± 0.7	1.48 ± 0.03*	9.3 ± 0.7	1.55 ± 0.03	4.6 ± 0.5	1.54 ± 0.03

Pilocarpine

	300 nM CBC (1st)		100 nM		300 nM		1 µM		3 µM		300 nM CBC (2nd)	
	TTP [s]	E_{MAX}	TTP [s]	E_{MAX}	TTP [s]	E_{MAX}	TTP [s]	E_{MAX}	TTP [s]	E_{MAX}	TTP [s]	E_{MAX}
M_1	6.1 ± 0.5	1.74 ± 0.03	13 ± 1	1.35 ± 0.03	13 ± 1	1.49 ± 0.03*	14 ± 1	1.60 ± 0.03*	14 ± 1	1.64 ± 0.03	6.3 ± 0.5	1.74 ± 0.03
M_2	4.3 ± 0.4	1.76 ± 0.03	10 ± 1	1.34 ± 0.03	9.8 ± 0.7	1.47 ± 0.03*	10 ± 1	1.59 ± 0.04*	10 ±1	1.64 ± 0.03	4.5 ± 0.4	1.77 ± 0.03
M_3	4.6 ± 0.4	1.79 ± 0.03	12 ± 1	1.34 ± 0.03	12 ± 1	1.50 ± 0.03*	12 ± 1	1.61 ± 0.03*	13 ± 1	1.66 ± 0.03	4.8 ± 0.4	1.79 ± 0.03
M_4	4.6 ± 0.4	1.62 ± 0.03	12 ± 1	1.35 ± 0.03	12 ± 1	1.47 ± 0.03*	13 ± 1	1.56 ± 0.03*	13 ± 1	1.59 ± 0.03	4.7 ± 0.4	1.63 ± 0.03
M_5	4.5 ± 0.4	1.54 ± 0.03	12 ± 1	1.29 ± 0.03	12 ± 1	1.41 ± 0.03*	13 ± 1	1.50 ± 0.03*	12 ± 1	1.53 ± 0.03	4.8 ± 0.4	1.54 ± 0.03

Parameters are derived from experiments shown in Fig. S1. Data are means ± S.E.M. from 3 independent experiments; TTM, time to reach the maximum; [*], different from the first (1st) stimulation by carbachol of the same concentration; [a], different from lower concentration by ANOVA and Tukey-Kramer post-test, $P<0.05$.

Table S4. Parameters of calcium level changes upon activation of individual muscarinic receptor subtypes by the agonists carbachol and xanomeline in Fig. 2 in the main manuscript.

	**hM_1**	**hM_2**	**hM_3**	**hM_4**	**hM_5**
**1st stimulation** (300 nM carbachol)					
Time needed to reach the maximum [s]	6.8 ± 0.7	6.2 ± 0.3	6.3 ± 0.7	6.9 ± 0.6	7.9 ± 0.7
Maximum (max_1) [Em ratio]	1.64 ± 0.05	1.67 ± 0.07	1.68 ± 0.09	1.53 ± 0.05	1.47 ± 0.04
**2nd stimulation** (10 µM xanomeline)					
Time needed to reach the maximum [s]	9.6 ± 1.7	29 ± 6	13 ± 6	17 ± 3	39 ± 3
Maximum [% max_1]	118 ± 3	45 ± 2	92 ± 6	103 ± 5	59 ± 4
Average value of steady signal in the time period of 30–60 min. [Em ratio]					
After 1 min xano	1.34 ± 0.06*	1.04 ± 0.03	1.12 ± 0.06*	1.18 ± 0.06*	1.01 ± 0.03
After 3 min xano	1.44 ± 0.12*	1.08 ± 0.05	1.16 ± 0.07*	1.19 ± 0.08*	1.02 ± 0.03
After 10 min xano	1.53 ± 0.09*	1.09 ± 0.05	1.18 ± 0.05*	1.31 ± 0.13*	1.03 ± 0.03
**3rd stimulation** (300 nM carbachol)					
Time needed to reach the maximum [s]					
After 1 min xano	n.a.	6.4 ± 0.3	14 ± 1[a]	7.1 ± 0.6	13 ± 1[a]
After 3 min xano	n.a.	7.4 ± 1.0	14 ± 1[a]	8.6 ± 0.9	13 ± 1[a]
After 10 min xano	n.a.	7.6 ± 0.3	15 ± 1[a]	n.a.	16 ± 1[a]
Maximum [% max_1]					
After 1 min xano	n.a.	99 ± 5	65 ± 4[a]	74 ± 3[a]	89 ± 5
After 3 min xano	n.a.	79 ± 4[a]	59 ± 4[a]	70 ± 3[a]	79 ± 4[a]
After 10 min xano	n.a.	61 ± 3[a]	55 ± 3[a]	n.a.	43 ± 2[a]

n.a., not applicable; *, different from basal level), [a], different from first stimulation, $P<0.05$ by Dunnett's test; Em ratio, fluorescence emission ratio. Data are average values ± S.E.M. from 6 independent measurements as those shown in Fig. 2 in the main manuscript.

Table S5. Parameters of calcium level changes upon activation of individual muscarinic receptor subtypes by the agonists carbachol, oxotremorine and pilocarpine in Fig. 3 in the main manuscript.

	**hM_1**	**hM_2**	**hM_3**	**hM_4**	**hM_5**
**1st stimulation** (300 nM carbachol)					
Time needed to reach the maximum [s]	6.4 ± 0.6	5.3 ± 0.5	5.5 ± 0.7	5.7 ± 0.6	5.9 ± 0.7
Maximum (max_1) [Em ratio]	1.73 ± 0.05	1.75 ± 0.07	1.77 ± 0.06	1.59 ± 0.05	1.53 ± 0.05
**2nd stimulation**					
Time needed to reach the maximum [s]					
Carbachol	6.4 ± 0.5	5.4 ± 0.5	5.6 ± 0.6	5.9 ± 0.6	6.0 ± 0.6
Oxotremorine	12 ± 1	9.6 ± 0.5	11 ± 1	10 ± 1	11 ± 1
Pilocarpine	16 ± 1	12 ± 1	15 ± 1	13 ± 1	15 ± 2
Maximum [Em ratio]					
Carbachol	1.92 ± 0.06	2.09 ± 0.05	2.08 ± 0.05	1.84 ±0.05	1.82 ± 0.05
Oxotremorine	1.65 ± 0.04	1.69 ± 0.03	1.68 ± 0.04	1.57 ± 0.04	1.60 ± 0.04
Pilocarpine	1.66 ± 0.04	1.68 ± 0.04	1.69 ± 0.04	1.64 ± 0.04	1.61 ± 0.05
Average value of steady signal in the time period of 30–60 min. [Em ratio]					
Carbachol	1.04 ± 0.01*	1.00 ± 0.01	1.00 ± 0.01	1.01 ± 0.01	1.00 ± 0.01
Oxotremorine	1.08 ± 0.02*	1.06 ± 0.01*	1.11 ± 0.02*	1.06 ± 0.02*	1.05 ± 0.02*
Pilocarpine	1.07 ± 0.02*	1.04 ± 0.01*	1.07 ± 0.03*	0.99 ± 0.01	1.09 ± 0.02*
**3rd stimulation** (300 nM carbachol)					
Time needed to reach the maximum [s]					
After carbachol	9.2 ± 0.5[a]	7.5 ±0.5[a]	6.1 ± 0.5	8.3 ± 0.5[a]	8.5 ± 0.5[a]
After oxotremorine	9.3 ± 0.5[a]	5.8 ± 0.5	5.8 ± 0.5	6.2 ± 0.5	6.3 ± 0.5
After pilocarpine	8.5 ± 0.5[a]	6.0 ± 0.5	5.6 ± 0.5	5.9 ± 0.5	n.a.[a]
Maximum [Em ratio]					
After carbachol	1.43 ± 0.03[a]	1.39 ± 0.04[a]	1.45 ± 0.03[a]	1.31 ± 0.03[a]	1.32 ± 0.03[a]
After oxotremorine	1.39 ± 0.03[a]	1.64 ± 0.03	1.60 ± 0.04	1.53 ± 0.04	1.52 ± 0.04
After pilocarpine	1.45 ± 0.04[a]	1.72 ± 0.05	1.76 ± 0.05	1.61 ±0.04	1.01 ± 0.01[a]

n.a., not applicable; *, different from basal level, [a], different from first stimulation, $P<0.05$ by Dunnett's test; Em ratio, fluorescence emission ratio. Data are average values ± S.E.M. from 3 independent measurements as those shown in Fig. 3 in the main manuscript.

Table S6. Parameters of calcium level changes induced by xanomeline at hM_1 through hM_4 receptors and effects of the antagonist NMS on delayed receptor activation in Fig. 4 in the main manuscript.

	**hM_1**	**hM_2**	**hM_3**	**hM_4**
	**1st stimulation (300 nM carbachol)**			
Time needed to reach the maximum [s]	4.2 ± 1.1	6.2 ± 0.8	4.0 ± 1.1	5.8 ± 0.9
Maximum (max1) [Em ratio]	1.28 ± 0.05	1.27 ± 0.07	1.29 ± 0.05	1.27±0.12
	**2nd stimulation (10 µM xano)**			
Time needed to reach the maximum [s]	14 ± 5	29 ± 7	15 ± 6	14 ± 3
Maximum (% max1) [Em ratio]	128 ± 9	98 ± 2	99 ± 3	96 ± 6
	**Application of 10 µM NMS**			
Inhibition effect (%)	98 ± 2	99 ± 1	100 ± 3	92 ± 3
Rise time after NMS application [s]	46 ± 8	n.a.	n.a.	136 ± 7
Maximum (max2) [Em ratio]	1.24 ± 0.05*	1.05 ± 0.06	1.00 ± 0.02	1.12 ± 0.02*

n.a., not applicable; *, different from basal level, $P<0.05$ by t-test, data from individual experiments paired; Em ratio, fluorescence emission ratio. Data are average values ± SEM from 6 independent measurements as those shown in Fig. 4 in the main manuscript.

Table S7. Parameters of calcium level changes induced by xanomeline in the presence of the antagonist NMS at M_1 through M_4 receptors in Fig. 5 in the main manuscript.

	**hM_1**	**hM_2**	**hM_3**	**hM_4**
**1st stimulation (300 nM carbachol)**				
Time needed to reach the maximum [s]	4.4 ± 0.3	5.6 ± 1.1	4.5 ± 0.6	5.8 ± 1.3
Maximum (max1) [Em ratio]	1.29 ± 0.05	1.28 ± 0.07	1.29 ± 0.09	1.34 ± 0.09
**2nd stimulation (10 µM xanomeline +10 µM NMS)**				
Inhibition effect of NMS [%]	4.5 ± 0.3*	n.a.	n.a.	n.a.
Rise time after NMS withdrawal [s]	27 ± 3	n.a.	240 ± 38	n.a.
Maximum (max2) [Em ratio]	1.12 ± 0.03*	1.03 ± 0.01	1.09 ± 0.02*	1.04 ± 0.02

n.a., not applicable; *, different from basal level, $P<0.05$ by Dunnett's test; Em ratio, fluorescence emission ratio. Data are average values ± S.E.M. From 6 to 8 independent measurements as those shown in Fig. 5 in the main manuscript.

Figures

Fig. S1 A

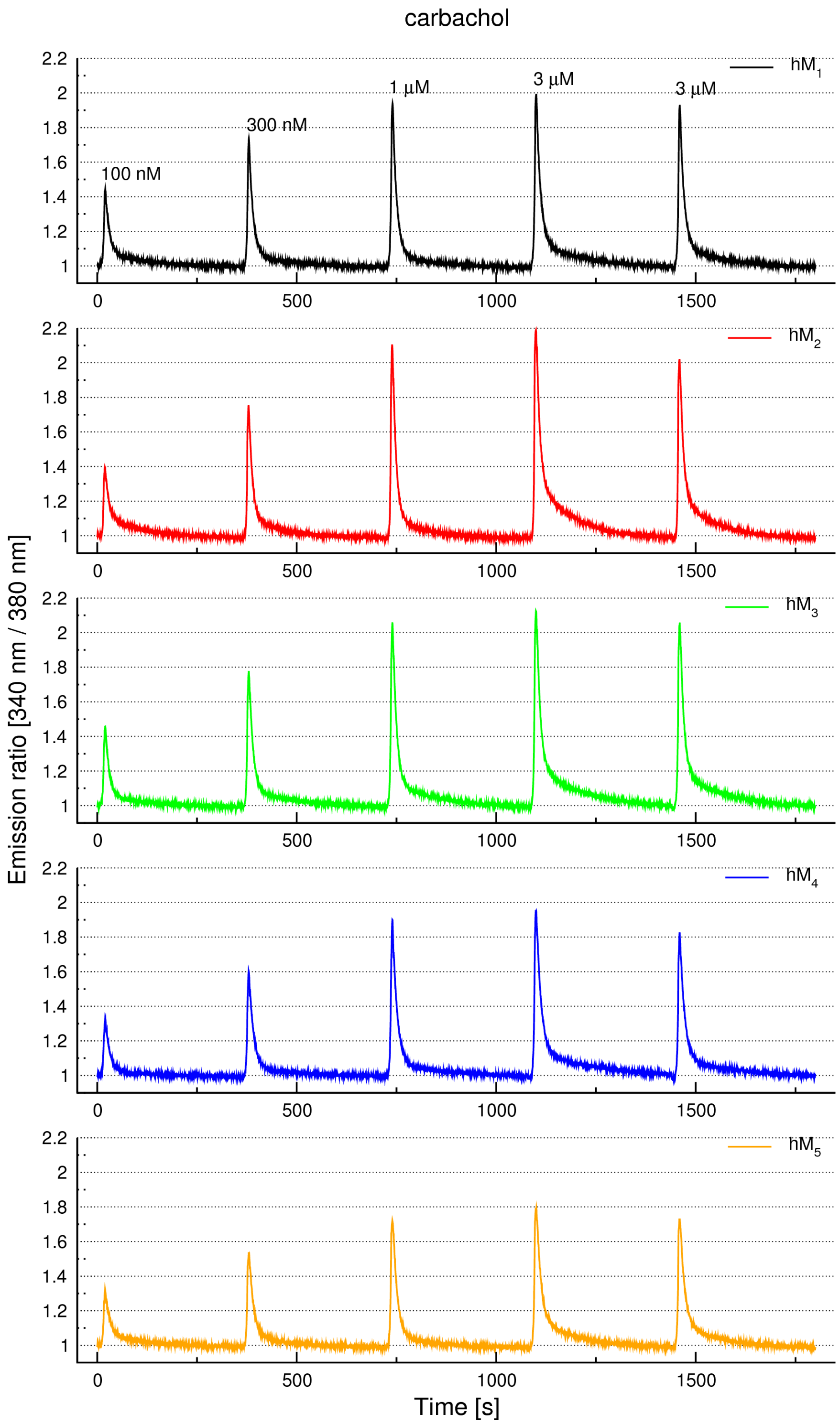

Fig. S1 B

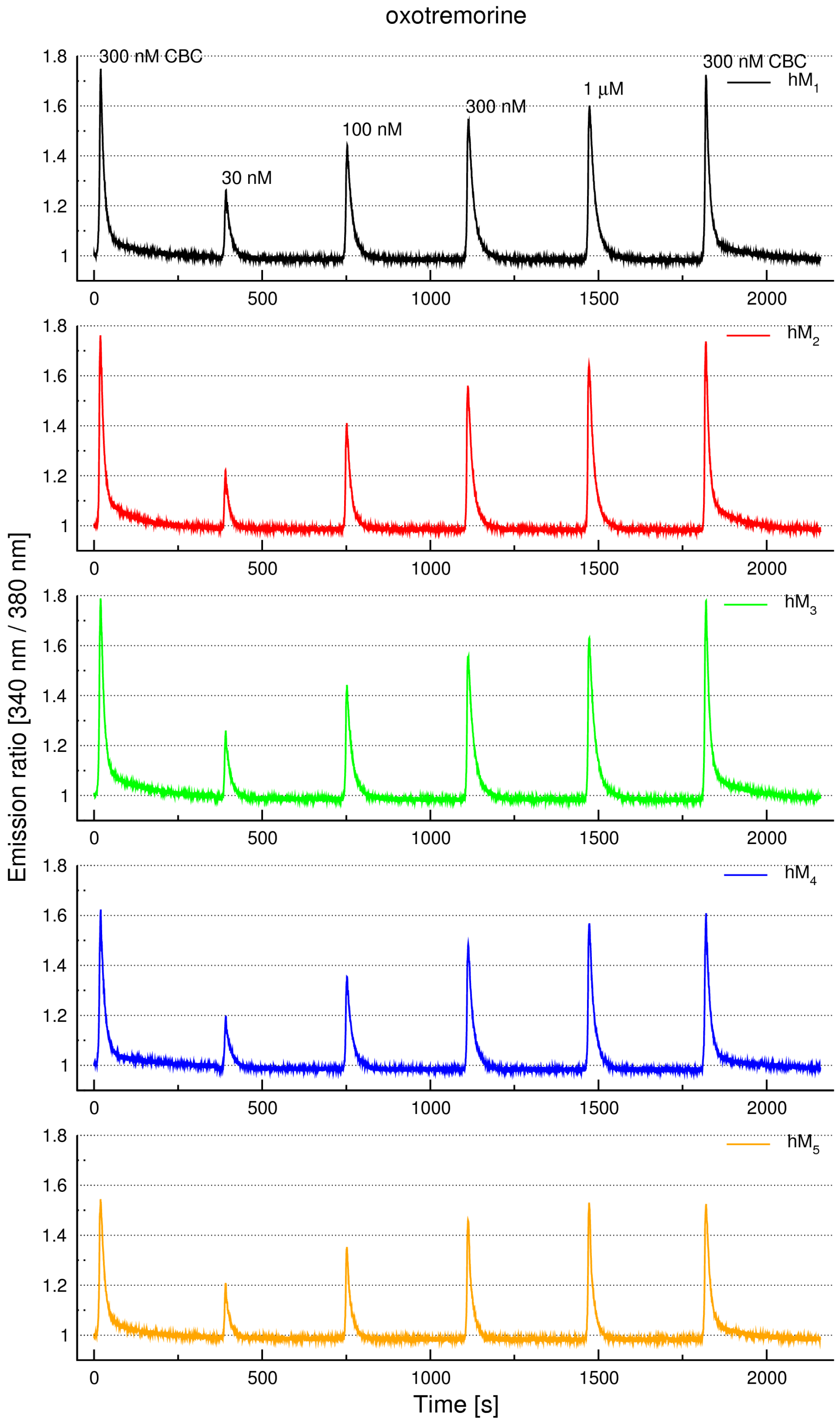

oxotremorine
300 nM CBC
30 nM
100 nM
300 nM
1 µM
300 nM CBC
hM1
hM2
hM3
hM4
hM5
1.8
1.6
1.4
1.2
1
0
500
1000
1500
2000
Emission ratio [340 nm / 380 nm]
Time [s]

SI Fig. 1C

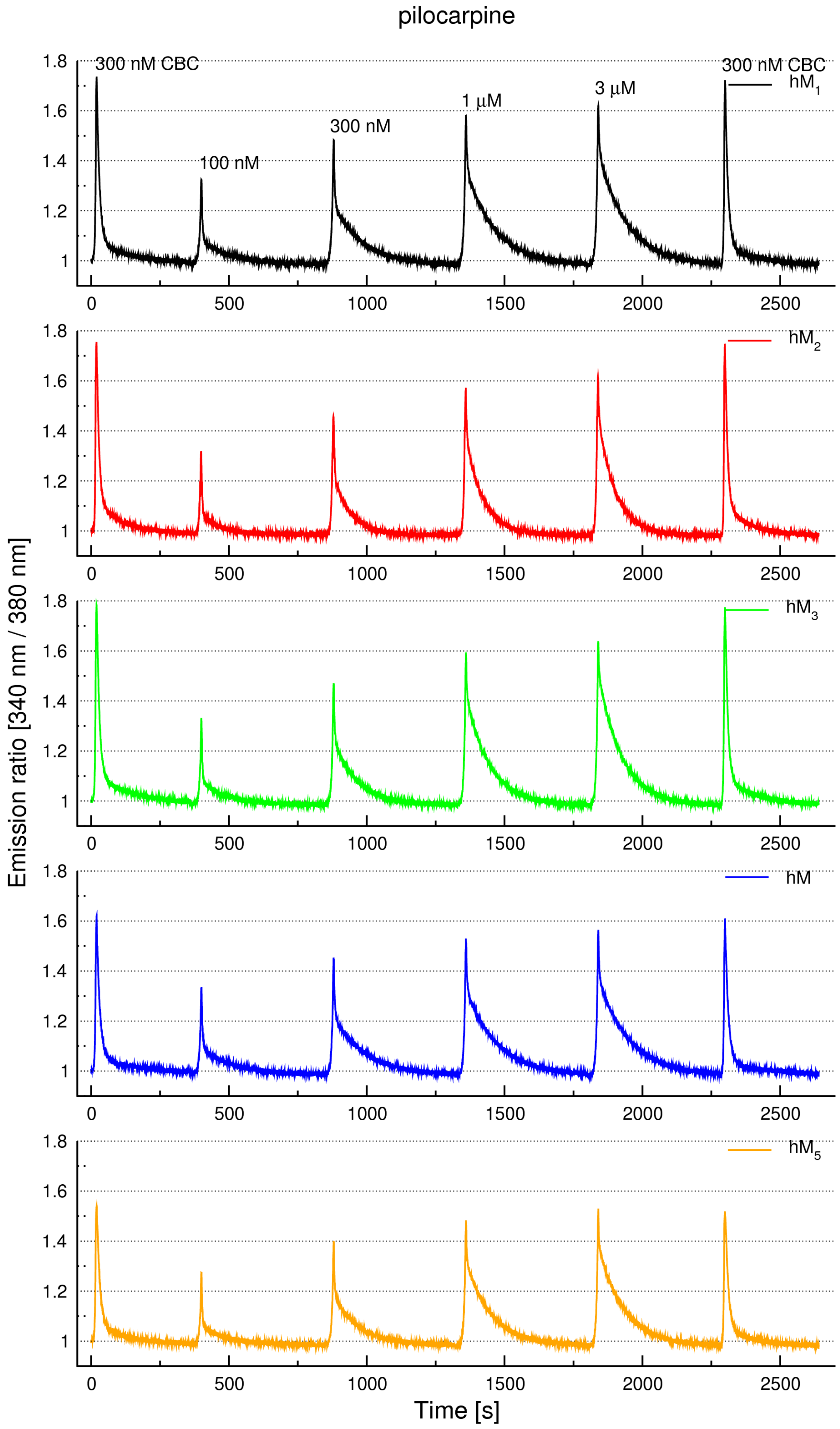
pilocarpine
300 nM CBC
100 nM
300 nM
1 µM
3 µM
300 nM CBC
hM1
hM2
hM3
hM
hM5
Emission ratio [340 nm / 380 nm]
Time [s]
1.8
1.6
1.4
1.2
1
0
500
1000
1500
2000
2500

Fig S1. Time courses of intracellular calcium response to acute treatment with the agonists carbachol, oxotremotine and pilocarpine

Cells were seeded, handled and loaded with Fura-2 as described in the main manuscript. Cells were repeatedly stimulated with increasing concentrations of carbachol (A), oxotremorine (B) or pilocarpine (C). **A:** After an initial 10-s period cells were stimulated with increasing concentrations of carbachol (100 nM, 300 nM, 1 µM and 3 µM) for 5 s. Then stimulation with 3 µM carbachol was repeated. Cells were perfused for 6 min between stimulations with KHB. **B:** After an initial 10-s period cells were stimulated with 300 nM carbachol (CBC) for 5 s then stimulated with increasing concentrations (30 nM, 100 nM, 300 nM and 1 µM) of oxotremorine for 10 s and then again with 300 nM carbachol for 5 s. Cells were perfused for 6 min between stimulations with KHB. **C:** After an initial 10-s period cells were stimulated with 300 nM carbachol (CBC) for 5 s then stimulated with increasing concetrations (100 nM, 300 nM, 1 µM and 3 µM) of pilocarpine for 20 s and then again with 300 nM carbachol for 5 s. Cells were perfused for 8 min between stimulations with KHB. Traces are averages from 8 cells from the representative experiment confirmed by 2 independent experiments. Signal variation (SD) among cells ranges from ± 0.021 at the base line to ± 0.088 at peaks. Parameters are summarized in Table S3.

Fig. S2

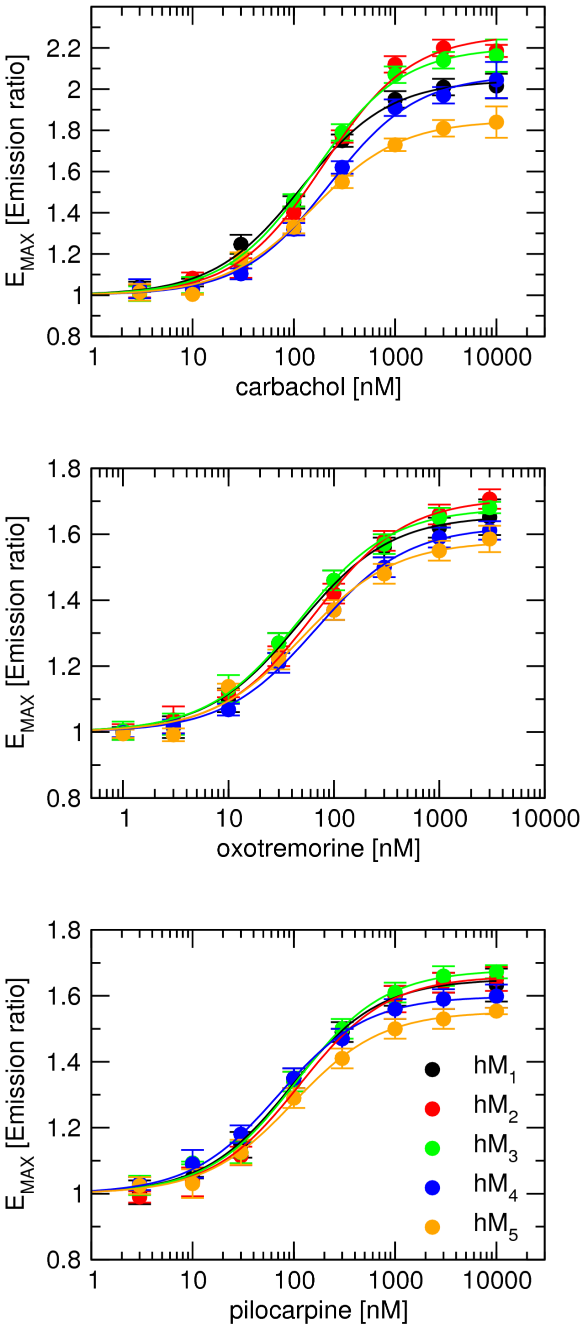

E_{MAX} [Emission ratio]
0.8
1
1.2
1.4
1.6
1.8
2
2.2
1
10
100
1000
10000
carbachol [nM]
E_{MAX} [Emission ratio]
0.8
1
1.2
1.4
1.6
1.8
1
10
100
1000
10000
oxotremorine [nM]
E_{MAX} [Emission ratio]
0.8
1
1.2
1.4
1.6
1.8
1
10
100
1000
10000
pilocarpine [nM]
hM_1
hM_2
hM_3
hM_4
hM_5

Fig. S2 Concentration response to acute treatment with the agonists carbachol, oxotremotine and pilocarpine.

Maximal effects (E_{MAX}) reached in intracellular calcium response were measured in the same experimental setup as in Fig. S1. E_{MAX} values are plotted against concentration of agonist carbachol (top), oxotremorine (middle) or pilocarpine (bottom). Data are means ± S.E.M. from 3 independent experiments. Eq. 1 of the main manuscript was fitted to the data. Parameters are summarized in Table 1 in the main manuscript.

Fig. S3 A

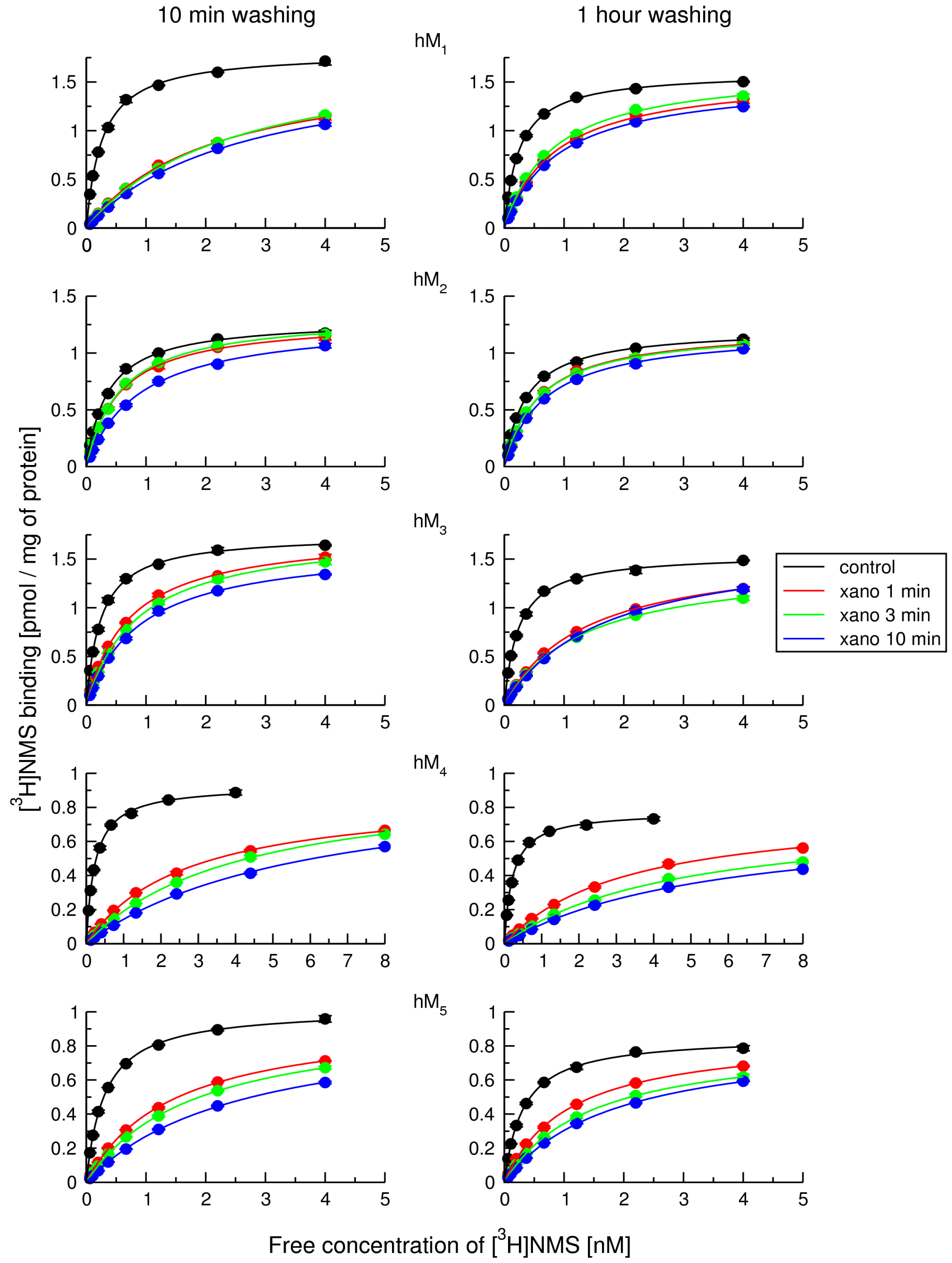
10 min washing
1 hour washing
hM1
hM2
hM3
hM4
hM5
control
xano 1 min
xano 3 min
xano 10 min
[3H]NMS binding [pmol / mg of protein]
Free concentration of [3H]NMS [nM]

Fig. S3 B

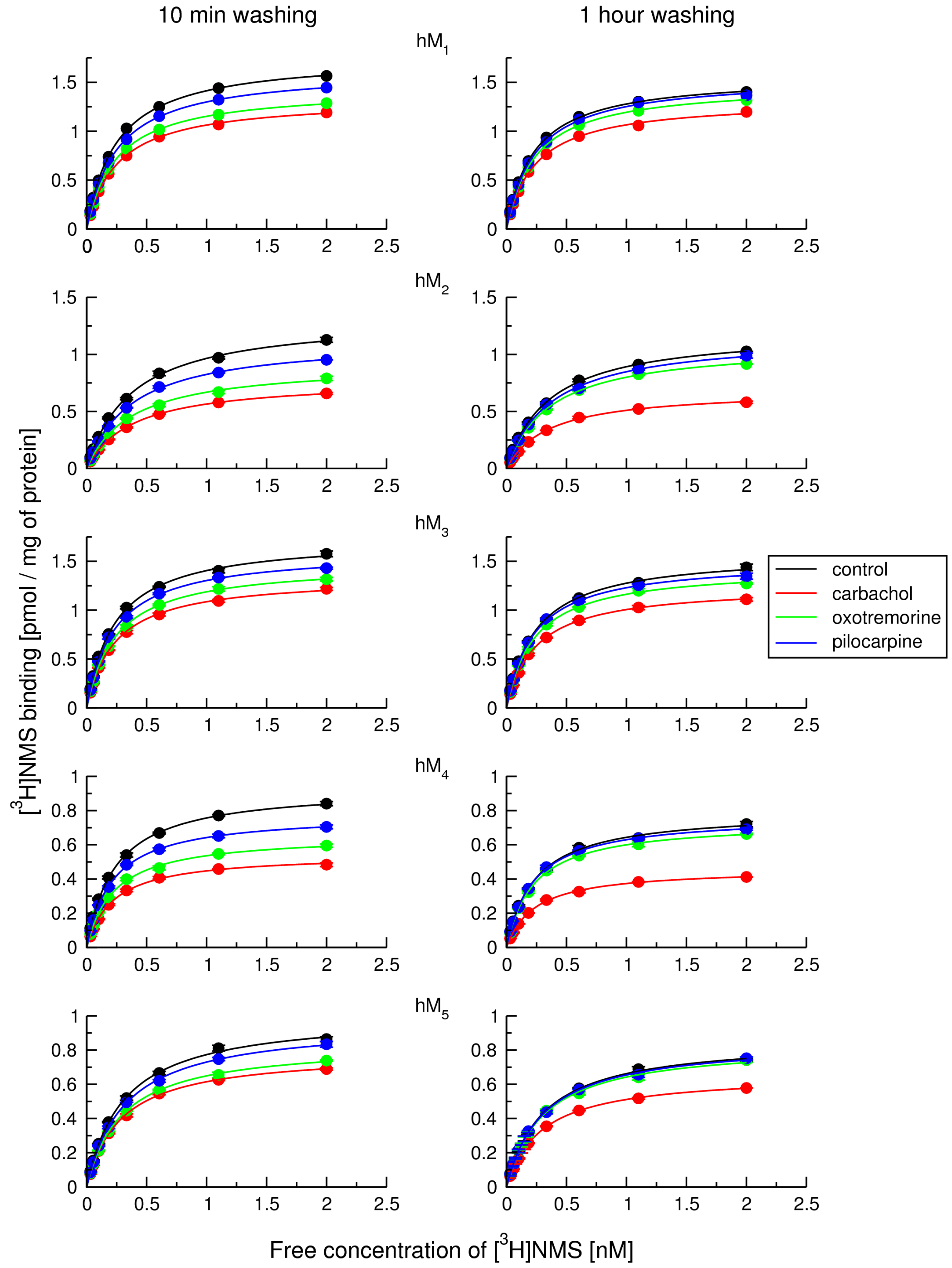
10 min washing
1 hour washing
hM1
hM2
hM3
hM4
hM5
control
carbachol
oxotremorine
pilocarpine
[3H]NMS binding [pmol / mg of protein]
Free concentration of [3H]NMS [nM]

Fig. S3. Saturation binding of [^{3}H]NMS to membranes from the cells treated with xanomeline. Intact cells expressing hM_1 to hM_5 receptors were exposed to 10 µM xanomeline (panel A) for 1 (red), 3 (green) or 10 min (blue) or for 10 min to agonists (panel B) 1 µM carbachol (red), 1 µM oxotremorine (green) and 3 µM pilocarpine (blue) or sham-treated (black) and washed with KHB for 10 min (left column) or 1 hour (right column) and membranes were prepared as described in Methods in main manuscript. Binding of the radiolabeled antagonist [^{3}H]NMS in concentrations ranging from 60 pM to 8 nM (panel A) or from 30 pm to 2 nM (panel B) to the membranes is plotted as pmol per mg of protein of specifically bound [^{3}H]NMS (ordinate) against concentration of free [^{3}H]NMS in nM (abscissa). Data are averages ± S.E.M. of 3 independent experiments performed in triplicates. Parameters of [^{3}H]NMS binding are summarized in Tables 2 and 3 in the main manuscript.

Fig. S4

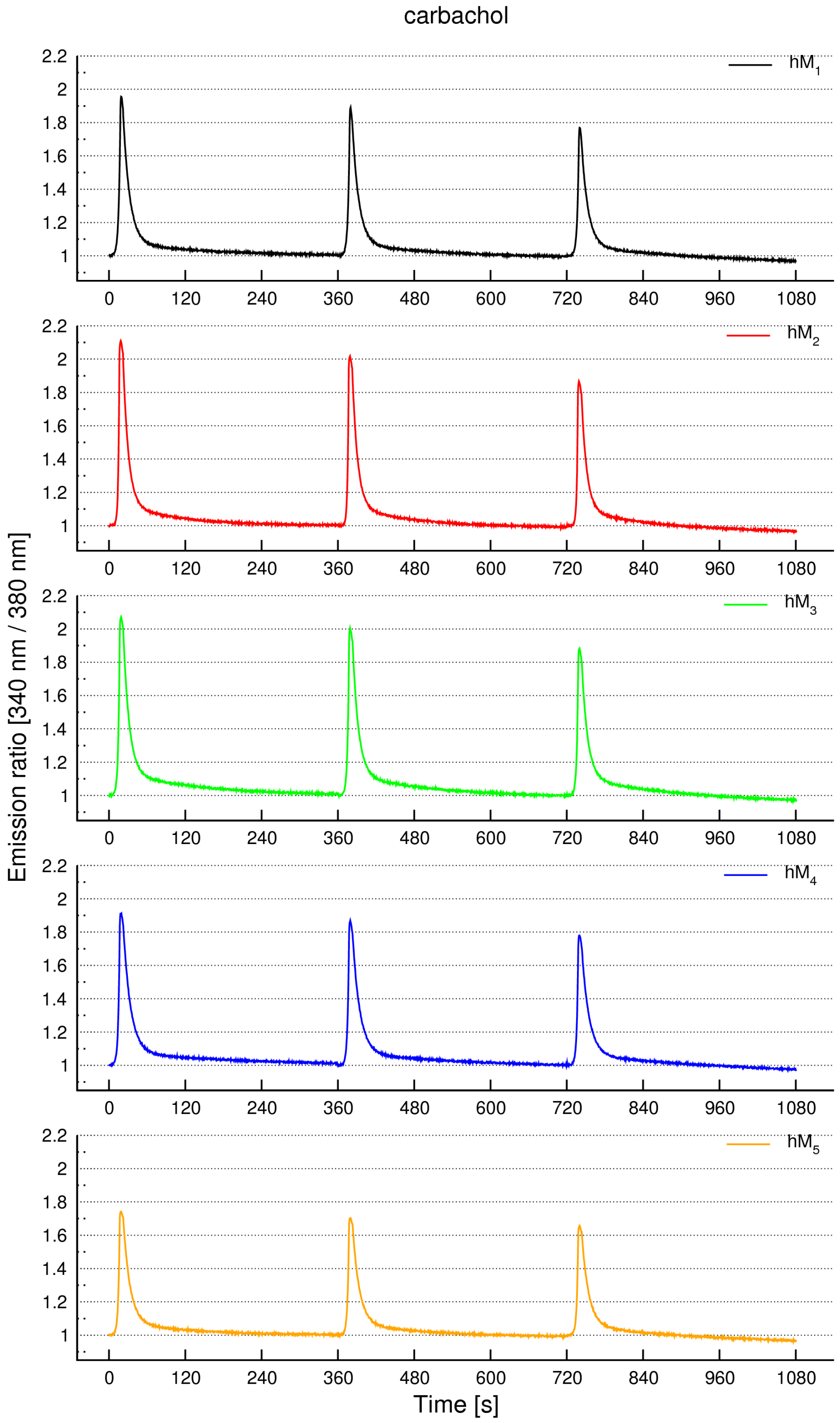

carbachol
hM1
hM2
hM3
hM4
hM5
Emission ratio [340 nm / 380 nm]
2.2
2
1.8
1.6
1.4
1.2
1
0
120
240
360
480
600
720
840
960
1080
Time [s]

Fig S4. Effects of changing the concentration of extracellular calcium.

Cells were seeded, handled and loaded with Fura-2 as described in the main manuscript. After an initial 10-s period cells were stimulated with 1 µM carbachol for 5 s, washed with KHB containing 1.3 µM $CaCl_2$ for 6 min, then washing was switched to KHB with $CaCl_2$ reduced to 0.65 µM then stimulated again with 1 µM carbachol for 5 s, washed with KHB containing 0.65 µM $CaCl_2$ for 6 min and then switched to calcium free KHB then stimulated again with 1 µM carbachol for 5 s and washed with calcium free KHB for final 6 min. Traces are averages from 10 to 12 cells from representative experiment confirmed by 2 independent experiments. Signal variation (SD) among cells ranges from ± 0.013 at the base line to ± 0.057 at peaks.
